# Four groups of type 2 diabetes contribute to the etiological and clinical heterogeneity in newly diagnosed individuals: An IMI DIRECT study

**DOI:** 10.1016/j.xcrm.2021.100477

**Published:** 2022-01-04

**Authors:** Agata Wesolowska-Andersen, Caroline A. Brorsson, Roberto Bizzotto, Andrea Mari, Andrea Tura, Robert Koivula, Anubha Mahajan, Ana Vinuela, Juan Fernandez Tajes, Sapna Sharma, Mark Haid, Cornelia Prehn, Anna Artati, Mun-Gwan Hong, Petra B. Musholt, Azra Kurbasic, Federico De Masi, Kostas Tsirigos, Helle Krogh Pedersen, Valborg Gudmundsdottir, Cecilia Engel Thomas, Karina Banasik, Chrisopher Jennison, Angus Jones, Gwen Kennedy, Jimmy Bell, Louise Thomas, Gary Frost, Henrik Thomsen, Kristine Allin, Tue Haldor Hansen, Henrik Vestergaard, Torben Hansen, Femke Rutters, Petra Elders, Leen t’Hart, Amelie Bonnefond, Mickaël Canouil, Soren Brage, Tarja Kokkola, Alison Heggie, Donna McEvoy, Andrew Hattersley, Timothy McDonald, Harriet Teare, Martin Ridderstrale, Mark Walker, Ian Forgie, Giuseppe N. Giordano, Philippe Froguel, Imre Pavo, Hartmut Ruetten, Oluf Pedersen, Emmanouil Dermitzakis, Paul W. Franks, Jochen M. Schwenk, Jerzy Adamski, Ewan Pearson, Mark I. McCarthy, Søren Brunak, IMI DIRECT Consortium

**Affiliations:** 1 Wellcome Centre for Human Genetics, University of Oxford, Oxford, UK; 2 Department of Health Technology, Technical University of Denmark, Kongens Lyngby, Denmark; 3 Novo Nordisk Foundation Center for Protein Research, Faculty of Health and Medical Sciences, University of Copenhagen, Copenhagen, Denmark; 4 C.N.R. Institute of Neuroscience, Padova, Italy; 5 Department of Genetic Medicine and Development, University of Geneva Medical School, Geneva, Switzerland; 6 Research Unit Molecular Endocrinology And Metabolism, Helmholtz Zentrum Muenchen, German Research Center for Environmental Health (GmbH), Neuherberg, Germany; 7 Affinity Proteomics, Science for Life Laboratory, School of Engineering Sciences in Chemistry, Biotechnology and Health, KTH Royal Institute of Technology, Solna, Sweden; 8 R&D Global Development, Translational Medicine & Clinical Pharmacology (TMCP), Sanofi-Aventis Deutschland GmbH, Frankfurt, Germany; 9 University of Lund, Clinical Sciences, Malmö, Sweden; 10 Department of Mathematical Sciences, University of Bath, Bath, UK; 11 University of Exeter Medical School, Exeter, UK; 12 The Immunoassay Biomarker Core Laboratory, Shool of Medicine, University of Dundee, Dundee, UK; 13 Research Centre for Optimal Health, Deparment of Life Sciences, University of Westminster, London, UK; 14 Section for Nutrition Research, Faculty of Medicine, Hammersmith Campus, Imperial College London, London, UK; 15 Novo Nordisk Foundation Center for Basic Metabolic Research, Faculty of Health and Medical Sciences, University of Copenhagen, Copenhagen, Denmark; 16 Department of Epidemiology and Biostatistics, Amsterdam Public Health Research Institute, Amsterdam UMC-location VUmc, Amsterdam, the Netherlands; 17 Department of General Practice, Amsterdam UMC-location VUmc, Amsterdam Public Health Research Institute, Amsterdam, the Netherlands; 18 Department of Cell and Chemical Biology, Leiden University Medical Center, Leiden, the Netherlands; 19 INSERM UMR 1283, CNRS UMR 8199, European Genomic Institute for Diabetes (EGID), Institut Pasteur de Lille, University of Lille, Lille University Hospital, Lille, France; 20 MRC Epidemiology Unit, University of Cambridge School of Clinical Medicine, Cambridge, UK; 21 Department of Medicine, University of Eastern Finland, Kuopio, Finland; 22 Institute of Cellular Medicine, Newcastle University, Newcastle, UK; 23 Diabetes Research Network, Royal Victoria Infirmary, Newcastle, UK; 24 Centre for Health, Law and Emerging Technologies (HeLEX), Faculty of Law, University of Oxford, Oxford, UK; 25 Translational and Clinical Research Institute, Faculty of Medical Sciences, Newcastle University, Newcastle, UK; 26 University of Dundee, Dundee, UK; 27 Eli Lilly Regional Operations GmbH, Vienna, Austria; 28 Lehrstuhl für Experimentelle Genetik, Technische Universität München, Freising-Weihenstephan, Germany; 29 Department of Biochemistry, Yong Loo Lin School of Medicine, National University of Singapore, 8 Medical Drive, Singapore 117597, Singapore; 30 Oxford Centre for Diabetes, Endocrinology and Metabolism, University of Oxford, Oxford, UK; 31 Oxford NIHR Biomedical Research Centre, Oxford University Hospitals NHS Foundation Trust, John Radcliffe Hospital, Oxford, UK

**Keywords:** type 2 diabetes, patient stratification, patient clustering, soft-clustering, archetypes, multi-omics, disease progression, glycaemic deterioration, precision medicine

## Abstract

The presentation and underlying pathophysiology of type 2 diabetes (T2D) is complex and heterogeneous. Recent studies attempted to stratify T2D into distinct subgroups using data-driven approaches, but their clinical utility may be limited if categorical representations of complex phenotypes are suboptimal.

We apply a soft-clustering (archetype) method to characterize newly diagnosed T2D based on 32 clinical variables. We assign quantitative clustering scores for individuals and investigate the associations with glycemic deterioration, genetic risk scores, circulating omics biomarkers, and phenotypic stability over 36 months. Four archetype profiles represent dysfunction patterns across combinations of T2D etiological processes and correlate with multiple circulating biomarkers. One archetype associated with obesity, insulin resistance, dyslipidemia, and impaired β cell glucose sensitivity corresponds with the fastest disease progression and highest demand for anti-diabetic treatment. We demonstrate that clinical heterogeneity in T2D can be mapped to heterogeneity in individual etiological processes, providing a potential route to personalized treatments.

## Highlights

• Soft clustering based on 32 phenotypes identified 4 quantitative archetypes
• These reflect different patterns of dysfunction across T2D etiological processes
• The four archetypes are different in disease progression, GRSs, and omics signals
• Some patients are dominated by one archetype, but many have etiological combinations

## Graphical abstract

**Figure:**
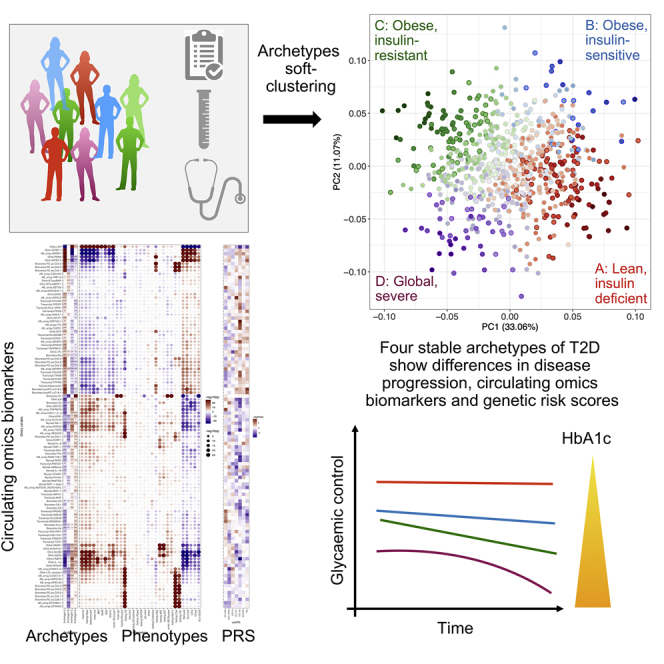

## Teaser

Wesolowska-Andersen et al. represent the clinical heterogeneity of newly diagnosed T2D as four quantitative archetype profiles reflecting patterns of dysfunction in disease etiological processes, rather than clustering individuals into categorical subgroups as attempted by others. The archetype profiles differ in genetic risk scores, disease progression, and circulating omics biomarkers.

## Introduction

Type 2 diabetes (T2D) is a complex, multifactorial disease characterized by hyperglycemia, which, at the level of the individual, is the consequence of dysfunction in several contributory disease processes, including adiposity, insulin resistance, and relative β cell failure. The clinical presentation and prognosis of T2D show considerable heterogeneity, and the same is true for rates of disease progression and individual response to anti-diabetic treatment. Stratification of disease based on patient characteristics at disease onset could help us better understand the mechanisms driving this heterogeneity and have clinical value in predicting the future course of disease and in guiding the development of tailored treatment plans.

Despite increasing knowledge about the different pathophysiologies that contribute to T2D predisposition, there is limited understanding of how these processes are related and how they drive differences in disease presentation and course. In the last decade, genome-wide association studies (GWASs) have characterized much of the genetic component of disease predisposition, with more than 400 genetic signals contributing to T2D susceptibility discovered to date and measurable genetic variation accounting for around 20% of overall variation in T2D predisposition.^1^ The molecular and pathophysiological mechanisms through which these variants act are beginning to emerge: they point to diverse processes (involving fat, muscle, liver, pancreatic islets, and brain) that contribute to an increased predisposition to T2D.^2^, ^3^, ^4^

One recently proposed approach to T2D stratification used a data-driven clustering method to subdivide patients with newly diagnosed diabetes into five subgroups based on individual measures of six clinical markers.^5^ The subgroups differed with respect to disease progression, use of anti-diabetic treatments, and risks of diabetes-related complications, as well as in the observed frequencies of several genetic variants predisposing individuals to T2D, indicating that the heterogeneity could be partly driven by the individuals’ genetic background. The five subgroups have been found to be reproducible in different cohorts, including diverse ethnicities.^6^, ^7^, ^8^ However, there has been considerable debate as to whether these subgroups represent distinct subtypes of T2D,^9^ particularly given that the genetic architecture of the disease (with most genetic risk attributable to common, widely shared genetic variants) and the impact of pervasive environmental risk factors appear more consistent with a model of continuous physiological dysfunction involving multiple molecular and pathophysiological processes in parallel (as in the proposed palette model).^10^ Indeed, one investigation demonstrated that the use of simple clinical phenotypes as continuous traits outperformed subgroup-based patient stratification in predicting progression and treatment responses,^6^ raising the question of whether subdivision of continuous multidimensional data into discrete clusters is clinically meaningful.

The aim of the present study was to characterize the complex phenotypic heterogeneity of T2D and its molecular features using a method that better captures the architecture of diabetes and aligns with the palette model. We investigated clinical, biochemical, and anthropometric measurements in newly diagnosed individuals from the IMI (Innovative Medicines Initiative) DIRECT (Diabetes Research on Patient Stratification) study. Instead of hard clusters defined with k-means clustering (which limit an individual to membership in a single cluster), we used a soft-clustering approach for patient stratification, which allows individuals to be members of more than one cluster. Soft clustering can reflect one or more concurrent pathophysiological processes in play and therefore align better with the available genetic and clinical data. This approach allowed us to measure the contribution of the etiological processes to the T2D phenotype using the quantitative clustering measures for individuals (we call these archetype scores) and, from the patterns that result, to identify subsets of individuals at the extreme end of these scores who have dysfunction in an extreme combination of etiological processes.

Access to rich phenotypic characterization and longitudinal follow-up within the IMI DIRECT cohort allowed us to explore the phenotypic determinants and clinical consequences of the etiological heterogeneity associated with the archetype scores, as well as to identify their contributing genetic factors and circulating biomarkers.

## Results

### Four archetypes of baseline T2D

We analyzed the baseline visit data from the newly diagnosed T2D cohort of the IMI DIRECT study, comprising of 726 participants (41% female) with complete data for 32 anthropometric, clinical, and biochemical phenotypes. All participants were diagnosed within two years before recruitment, were on lifestyle and/or metformin treatment only, and had HbA_1c_ < 60.0 mmol/mol (<7.6%) within previous the three months. Mean age at baseline visit was 62 years, mean BMI was 30.4 kg/m^2^, and mean HbA_1c_ was 46.4 mmol/mol (Table 1).

**Table 1:** Descriptive characteristics of the full cohort and differences among the groups with extreme archetype scores at baseline

Phenotype	Full cohort	A	B	C	D	MIX	p value
Number of individuals	726	103	22	84	45	472	NA
XX/XY genotype (%)	298/428 (41%)	46/57 (45%)	8/14 (36%)	30/54 (36%)	21/24 (47%)	193/279 (41%)	0.9
Age (years)	61.97 (8.03)	64.78 (6.08)	62.68 (8.52)	60.88 (7.43)	59.16 (8.07)	61.79 (8.34)	0.00016
BMI (kg/m^2^)	30.44 (4.97)	25.42 (2.55)	30.99 (4.85)	33.47 (4.59)	32.91 (4.61)	30.74 (4.69)	2.2E−35
WHR (m/m)	0.96 (0.08)	0.91 (0.07)	0.97 (0.07)	1.00 (0.07)	0.99 (0.08)	0.97 (0.08)	3.6E−11
BSA (m^2^)	2.07 (0.23)	1.87 (0.19)	2.15 (0.18)	2.17 (0.21)	2.12 (0.21)	2.09 (0.22)	1.2E−19
Fasting C-peptide (pmol/L)	1,074.99 (392.22)	706.27 (184.29)	683.27 (223.54)	1,627.56 (417.47)	1,318.58 (315.72)	1,052.15 (295.45)	3.3E−60
Fasting HbA_1c_ (mmol/mol Hb)	46.41 (5.71)	44.48 (4.19)	45.64 (5.70)	44.88 (4.73)	56.04 (5.75)	46.22 (5.27)	3.8E−20
Fasting glucose (mmol/L)	7.1 (1.39)	6.83 (0.88)	4.15 (1.30)	6.91 (0.85)	9.47 (1.45)	7.10 (1.14)	6.02E−30
Fasting insulin (nmol/L)	104.64 (67.23)	44.17 (17.90)	54.49 (27.58)	209.32 (86.40)	143.51 (64.08)	97.84 (45.75)	2.1E−67
Fasting HDL-C (mmol/L)	1.19 (0.38)	1.52 (0.42)	0.66 (0.29)	1.09 (0.32)	1.04 (0.27)	1.17 (0.34)	8.1E−25
Fasting LDL-C (mmol/L)	2.34 (0.96)	2.86 (1.04)	1.19 (0.61)	2.27 (0.87)	2.53 (0.81)	2.28 (0.92)	1.7E−13
Fasting TG (mmol/L)	1.51 (0.8)	1.24 (0.48)	0.71 (0.41)	1.79 (1.24)	2.13 (0.86)	1.50 (0.70)	2.8E−16
Fasting ALT (U/L)	26.43 (14.03)	21.30 (8.07)	15.95 (9.88)	32.73 (18.50)	36.38 (17.34)	25.96 (12.98)	2.4E−16
Fasting AST (U/L)	25.61 (10.87)	26.04 (8.82)	15.95 (5.61)	27.93 (12.55)	33.44 (17.00)	24.81 (9.85)	1.1E−11
Fasting cholesterol (mmol/L)	4.23 (1.13)	4.95 (1.10)	2.19 (10.90)	4.17 (0.93)	4.54 (0.94)	4.15 (1.06)	6.7E−19
Fasting creatinine (umol/L)	75.19 (17.38)	79.45 (13.62)	42.00 (19.10)	77.92 (17.57)	78.44 (17.84)	75.01 (16.32)	1.5E−9
Fasting UCPCR (nmol/mmol)	3.38 (2.16)	2.70 (1.63)	2.61 (1.05)	5.00 (3.44)	3.11 (1.56)	3.31 (1.92)	3.1E−10
Fasting UCpep (nmol/L)	29.95 (22.94)	19.96 (18.63)	28.19 (19.78)	49.30 (31.16)	28.70 (19.84)	28.90 (20.49)	3.6E−15
Fasting UCreatinine (mmol/L)	9.72 (5.87)	8.00 (5.55)	11.29 (7.08)	11.56 (6.31)	10.31 (5.99)	9.64 (5.69)	4.6E−4
MMTT 120 min glucose (mmol/L)	8.72 (2.78)	7.52 (2.12)	5.11 (2.12)	8.32 (1.93)	13.88 (2.18)	8.73 (2.47)	3.4E−32
MMTT 120 min insulin (nmol/L)	451.03 (350.52)	222.18 (127.36)	195.48 (135.96)	947.31 (598.67)	474.30 (203.36)	422.34 (241.77)	1.7E−44
Mean glucose (nmol/L)	9.34 (2.01)	8.53 (1.46)	6.15 (1.42)	9.03 (1.25)	12.87 (1.84)	9.37 (1.80)	6.8E−32
Mean insulin (pmol/L)	458.01 (276.45)	250.89 (104.02)	270.44 (128.76)	936.62 (386.54)	424.44 (158.74)	429.97 (181.93)	1.8E−57
Basal insulin secretion rate (pmol min^−1^ m^−2^)	135.87 (47.45)	93.43 (23.39)	87.24 (24.45)	202.81 (51.81)	165.59 (42.28)	132.65 (35.82)	4.5E−58
Total insulin secretion (nmol m^−2^)	44.14 (14.37)	35.98 (9.86)	28.68 (8.77)	64.86 (13.84)	41.33 (14.76)	43.23 (1.64)	2.3E−43
Glucose sensitivity (pmol min^−1^ m^−2^ L mmol^−1^)	83.67 (55.01)	79.80 (50.91)	70.86 (46.90)	135.46 (69.91)	34.50 (17.20)	80.58 (48.79)	6.7E−29
Rate sensitivity (pmol m^−2^ L mmol^−1^)	1,115.08 (1,044.94)	950.03 (925.31)	773.00 (696.38)	1,715.03 (1,511.39)	852.24 (576.16)	1,085.32 (978.29)	1.0E−45
Potentiation fraction ratio (no unit)	1.41 (0.57)	1.76 (0.72)	1.15 (0.40)	1.29 (0.45)	1.13 (0.20)	1.39 (0.55)	1.5E−9
Stumvoll	5.51 (2.71)	8.33 (1.22)	7.61 (1.44)	2.27 (3.35)	3.52 (1.63)	5.56 (2.11)	2.7E−59
Matsuda	2.94 (2.21)	5.32 (2.59)	7.23 (4.12)	1.15 (0.38)	1.51 (0.61)	2.67 (1.52)	5.0E−71
2hOGIS (mL min^−1^ m^−2^)	297.17 (69.03)	334.83 (52.17)	495.05 (127.13)	246.21 (42.96)	231.64 (27.28)	295.05 (51.11)	6.4E−49
Basal insulin clearance (L min^−1^ m^−2^)	1.61 (1.02)	2.45 (2.02)	1.65 (0.35)	1.05 (0.28)	1.28 (0.39)	1.55 (0.69)	2.6E−47
Insulin clearance (L min^−1^ m^−2^)	0.93 (0.3)	1.29 (0.33)	0.96 (0.26)	0.62 (0.15)	0.86 (0.20)	0.92 (0.25)	1.2E−49

All phenotypes are summarized as mean and SD in parentheses. Differences among the groups were tested with the Kruskal-Wallis test and adjusted for multiple testing with the Benjamini-Hochberg procedure to reduce the false discovery rate (FDR). UCPCR, urine C-peptide/creatinine ratio; UCpep, urine C-peptide; UCreatinine, urine creatinine.

**Figure 1:**
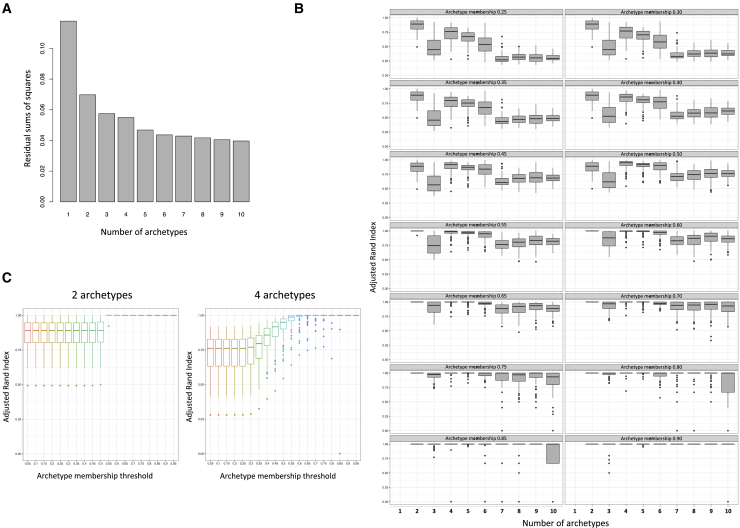
Archetype stability as evaluated using the following approaches (A) Minimized residual sum of squares (RSS) for k number of archetypes ranging from 1 to 10 in a scree plot was assessed first. The screen plot was based on RSS from the best model out of 100 restarts of the archetype algorithm for each k and showed a plateau in the drop in intra-cluster variance at k4 or k5. (B) Stability of the k archetypes was then assessed by a randomized subsampling of 90% of the original dataset repeated 100 times and compared to the original subgroups using the adjusted Rand index. Simultaneously, we evaluated the stability of the archetypes at archetype membership cutoffs ranging from 0 to 1 in intervals of 0.05. The most stable solution was k2, irrespective of membership threshold, followed by k4, which reached a median adjusted Rand index > 0.75 at threshold 0. (C) Stability of the solution with two and four archetypes across the full range of tested archetype membership thresholds. Altogether, these analyses showed that four archetypes had the lowest RSS while showing high stability after randomization. The subgroup stability increased with an increasing membership threshold and plateaued at 0.6, wherefore this threshold was used as the cutoff for the extreme archetype inclusion. Whiskers in (B) and (C) correspond to the largest and smallest value no further than 1.5 IQR (inter quartile range) from the hinge.

**Figure 2:**
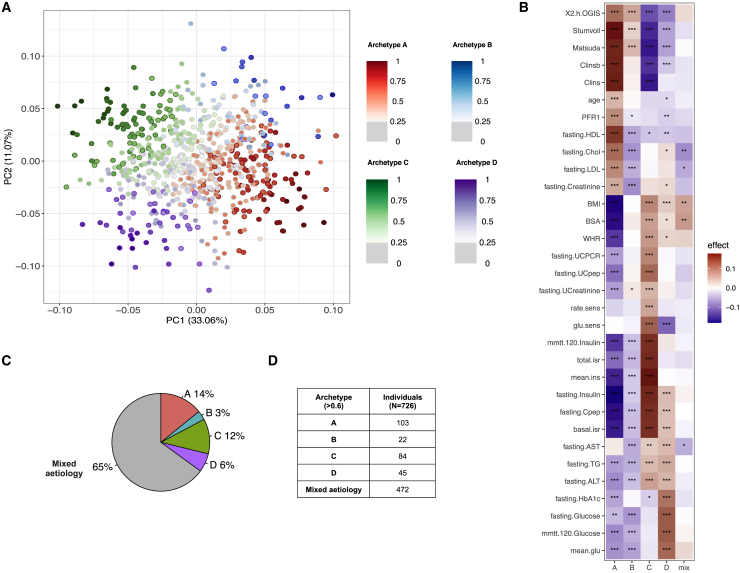
Clinical characteristics of the four archetypes, and groups with archetype scores identified at the extremes of the baseline phenotype spectrum (A) Representation of the baseline phenotype spectrum of newly diagnosed T2D projected in 2 dimensions following principal-component analysis. Each point represents an individual, and the four archetypes are colored and marked as subgroups A–D. The strength of the colors represents the level of archetype membership, with individuals shown in a lighter color representing a mixed phenotype with no clearly dominating archetype. (B) Summary of the 32 clinical variables used for the characterization of the baseline T2D phenotypic space. All variables were rank-normally transformed, and for each group with extreme archetype scores and each variable, the heatmap shows the significance level of the difference between the group and the remaining individuals from the study, as from a Mann-Whitney U test. The color of the heatmap reflects the directionality and magnitude of the test estimate, with red indicating higher values and blue indicating lower values characteristic of the given group. (C) Pie chart showing the percentage of individuals belonging to each of the four groups with extreme archetype scores and in the mixed etiology group. (D) Table of the number of individuals represented in each of the four groups with extreme archetype scores and in the mixed etiology group. Values statistically different from zero are marked as ∗p < 0.05, ∗∗p < 0.01, and ∗∗∗p < 0.001.

Instead of applying a hard-clustering approach to impose discrete clusters, we used the soft-clustering method of archetypes^11^^,^^12^ to uncover the baseline T2D etiological processes that contribute to disease heterogeneity. The archetype analysis identifies extreme observations within a multivariate dataset and subsequently represents individuals in the phenotype spectrum as convex combinations of these extreme observations. Our analysis identified four stable baseline archetypes named A, B, C, and D (Figure 1), which resulted in four quantitative archetype scores defined for individuals, such that the four archetype scores summed to 1 for an individual. Most individuals (n = 472) were located in the middle of the phenotype distribution with moderate contributions from two or more archetypes, likely representing dysfunction in multiple etiological processes (mixed etiology group) (Figure 2A). To identify the phenotypes characterizing the archetypes, we focused on the individuals at the extremes of the distribution (defined by archetype membership > 0.6, n = 254 [35%]) with higher degrees of dysfunction across combinations of etiological processes captured by the four archetype scores (Figures 2B–2D).

There were 103 individuals with extreme values for the archetype A score. They were characterized by low BMI, older age, high insulin sensitivity, and high cholesterol. Individuals with extreme values for archetype B, C, and D scores were obese on average, but individuals with an extreme archetype B score (n = 22) were insulin sensitive and associated with favorable lipid profiles and low fasting creatinine levels. Extreme values for the archetype C score (n = 84) were associated with insulin resistance (low 2 h oral glucose insulin sensitivity [2hOGIS], Stumvoll, and Matsuda indices, as well as high fasting and mixed-meal tolerance test [MMTT] insulin levels). Individuals with extreme values for the archetype D score (n = 45), in addition to obesity and insulin resistance, had the worst glucose control and low glucose sensitivity, indicating β cell dysfunction. In addition, extreme values for both the archetype C and the archetype D scores were associated with high levels of triglycerides (TGs) and the liver enzymes ALT and AST, indicating dyslipidemia (Figure 2B; Table 1; Table S1A). Highly similar results were obtained when we investigated associations with the quantitative archetype scores, with all individuals contributing to the analysis (Figures S3A and S3B; Table S1B). Hence, we investigated the associations with the quantitative archetype scores in all subsequent analyses to increase the statistical power for discovery.

### Archetypes associated with differences in genetic risk of T2D

**Figure 3:**
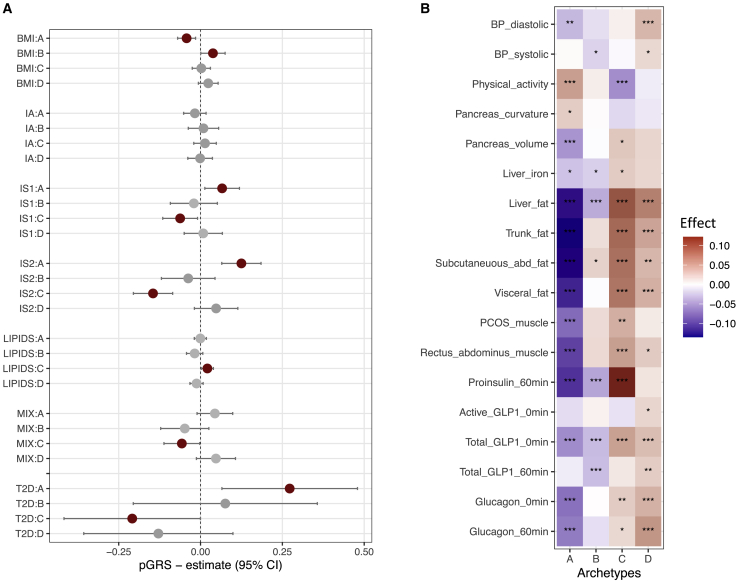
Archetype associations with genetic risk scores and additional clinical variables (A) Associations of partitioned genetic risk scores or T2D genetic risk scores with archetype scores. Statistically significant results (p < 0.05) from linear regression are shown in red. (B) Associations of clinical variables available for a subset of the cohort or only collected at the baseline visit. These are not used in the clustering of the baseline T2D phenotypic space. All variables were rank-normally transformed, and for each archetype score and each variable, the heatmap shows the significance level of the association test using linear regression. The color of the heatmap reflects the directionality and magnitude of the test estimate, with red indicating positive and blue indicating negative associations. Values statistically different from zero are marked as ∗p < 0.05, ∗∗p < 0.01, and ∗∗∗p < 0.001.

To elucidate the primary factors driving the etiological processes that contribute to the archetypes, we investigated their genetic contribution. We calculated genetic risk scores (GRSs) for T2D, as well as six partitioned genetic risk scores (pGRSs) for loci classified as involved in reduced insulin secretion with high proinsulin (IS1) and low proinsulin (IS2), insulin action (IA), adiposity (BMI), dyslipidemia (LIPID),^13^ and mixed features (MIX), and evaluated whether these pGRSs differed among archetypes (Figure 3A; Figure S4; Table S2). We observed a significant difference in the overall T2D-GRS for archetype A and C scores (β_A_ = 0.3, p_A_ = 0.010; β_C_ = −0.2, p_C_ = 0.050), which implies that a higher archetype A score was nominally associated with greater overall genetic predisposition for T2D, whereas a higher archetype C score was associated with lower overall risk. We also found that the archetype A score was significantly associated with two previously reported insulin secretion pGRSs; i.e., those individuals with a high archetype A score were genetically predisposed to lower β cell function (IS1: β_A_ = 0.07, p_A_ = 0.02; IS2: β_A_ = 0.1, p_A_ = 4.9 × 10^−5^), whereas we observed the opposite direction of associations for the archetype C score (IS1: β_C_ = −0.06, p_C_ = 0.02; IS2: β_C_ = −0.2, p_C_ = 2.4 × 10^−6^). Finally, we observed that the archetype B score was associated with higher BMI-pGRS values, as opposed to the archetype A score, which was associated with lower BMI-pGRS values (β_A_ = −0.04, p_A_ = 2.4 × 10^−3^; β_B_ = 0.04, p_B_ = 0.05). Overall, this led us to conclude that the likely primary processes driving the phenotypic differences among the archetypes was insulin deficiency associated with a higher archetype A score, obesity associated with a higher archetype B score, and insulin resistance associated with a higher archetype C score. The genetic evidence for archetype D was inconclusive, but given the associations with the severest phenotypes, i.e., younger age, obesity, and insulin resistance, coupled with low glucose sensitivity, we described this archetype as global severe. For the other archetypes, we chose the descriptions lean and insulin deficient (archetype A), obese and insulin sensitive (archetype B), and obese and insulin resistant (archetype C).

### Archetypes associated with differences in baseline adiposity, physical activity, and hormone levels

We further explored associations between archetypes and additional clinical variables (Figure 3B; Table S3). Among the most notable differences, we observed that higher scores for archetype A (lean and insulin deficient) were linked to higher physical activity (p_A_ = 3.6 × 10^−7^), lower subcutaneous and visceral adiposity (p_A_ < 5.5 × 10^−21^), and lower stimulated proinsulin, glucagon, and total GLP-1 levels (p_A_ < 4.0 × 10^−8^). In contrast, higher scores for archetype C (obese and insulin resistant) were associated with higher levels of these hormones (p_C_ < 0.007), as well as higher MRI-measured fat levels (p_C_ < 7.9 × 10^−9^). High scores for both archetype C (obese and insulin resistant) and archetype D (global severe) were characterized by higher liver fat (p_C_ = 5.6 × 10^−16^ and p_D_ = 4.5 × 10^−10^), and a high archetype D score was associated with higher diastolic blood pressure (p_D_ = 8.22 × 10^−5^). A high score for archetype B (obese and insulin sensitive) was associated with lower liver fat (p_B_ = 8.1 × 10^−5^), stimulated proinsulin, and fasting and stimulated GLP-1 levels (p_B_ < 1.7 × 10^−4^).

### Archetypes associated with differences in disease progression rates

**Figure 4:**
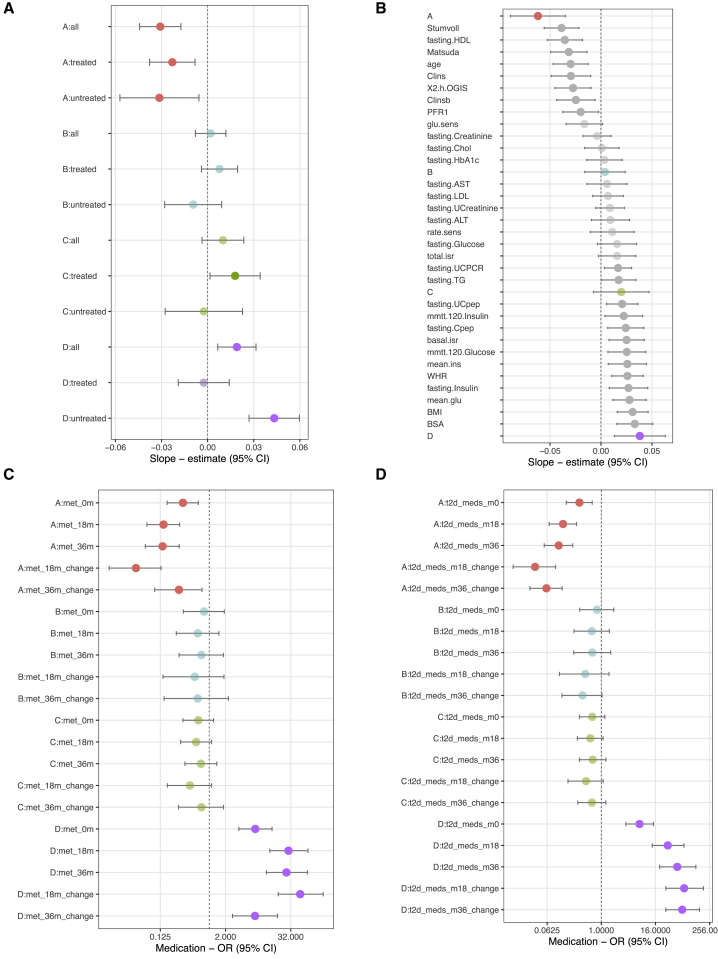
Association between archetypes and disease progression as defined by the slope of the HbA_1c_ increase and by glucose-lowering medication during the 36-month study period (A) T2D disease progression assessed as HbA_1c_ slopes as dependent variables and archetype scores as independent variables. The analysis was divided into all individuals, untreated individuals, and individuals treated with glucose-lowering medication at baseline for each archetype. (B) Ability of individual phenotypes to predict T2D disease progression. Combinations of phenotypes constituting archetypes A and D had the highest power to predict disease progression. (C) Forest plot showing the odds ratios between archetype scores and individuals receiving metformin treatment or increasing their metformin treatment (change) during the study period. (D) Forest plot showing the odds ratios between archetype scores and individuals receiving glucose-lowering treatment or increasing their treatment (change) during the study period. Error bars represent 95% confidence intervals. Statistically significant results (p < 0.05) from linear or logistic regression are shown in opaque colors.

To assess the clinical relevance of the archetypes, we evaluated whether the four archetypes reflect differences in disease progression by analyzing their association with slopes of HbA_1c_ change over time for 696 individuals with at least two HbA_1c_ measurements. We observed that a high score for archetype A (lean and insulin deficient) was associated with the slowest progression (β_A_ = −0.03, p_A_ = 7.8 × 10^−6^) and a high score for archetype D (global severe) was associated with the fastest progression (β_D_ = 0.02, p_D_ = 2.8 × 10^−3^). Because the HbA_1c_ values are affected by any administered glucose-lowering medication, we also performed this analysis stratified by medication use: 346 participants on lifestyle treatment only and 350 participants receiving T2D medication at baseline (Figure 4A; Table S4). The fastest progression was observed for a high archetype D score among participants on lifestyle treatment (β_D_lifestyle_ = 0.04, p_D_lifestyle_ = 3.4 × 10^−7^) (Table S4), but it was not associated with progression among the individuals treated with metformin (β_D_T2Dtreatment_ = −0.003, p_D_T2Dtreatment_ = 0.8). We compared the archetype score associations with disease progression to the corresponding performance of the single phenotypes and discovered that the combinations of etiological processes defining archetypes A and D had the highest power to predict disease progression (Figure 4B).

As an alternative measure of disease progression, we investigated whether the archetypes were associated with differences in likelihood of receiving glucose-lowering medication during the study period (Figures 4C and 4D; Table S5). A higher score for archetype D (global severe) was associated with the highest risk of being on glucose-lowering medication at all time points. At baseline, there was already a significant association (odds ratio [OR] at month 0: OR_M0_ = 7.1, p_M0_ = 5.4 × 10^−8^), but this was more pronounced at the follow-up visits (OR_M18_ = 30.0, p_M18_ = 2.4 × 10^−16^; OR_M36_ = 48.8, p_M36_ = 31.7 × 10^−16^). In contrast, a higher score for archetype A (lean and insulin deficient) was associated with the lowest likelihood of receiving glucose-lowering medication at all time points (OR_M0_ = 0.3, p_M0_ = 9.4 × 10^−4^; OR_M18_ = 0.1, p_M18_ = 4.5 × 10^−8^; OR_M36_ = 0.1, p_M36_ = 6.9 × 10^−8^). In addition, we examined whether the archetypes were associated with participants requiring an increase in dosage or starting a new anti-diabetic treatment, indicating that their blood glucose control worsened (Figure 4D). Again, we observed that a higher archetype D score was associated with the highest risk (OR_changeM18_ = 69.4, p_changeM18_ = 3.7 × 10^−18^; OR_changeM36_ = 62.8, p_changeM36_ = 5.2 × 10^−21^) and a higher archetype A score was associated with the lowest risk (OR_changeM18_ = 0.03, p_changeM18_ = 9.2 × 10^−10^; OR_changeM36_ = 0.06, p_changeM36_ = 2.2 × 10^−11^). This was true for individual groups of anti-diabetic medication administered (Table S5). Altogether, a higher score for archetype D (global severe) was associated with the fastest disease progression, in particular within the lifestyle-treated subset, suggesting that early identification of individuals with dysfunction in the associated etiological processes would be beneficial.

### Archetypes were defined by distinct circulating omics signatures

**Figure 5:**
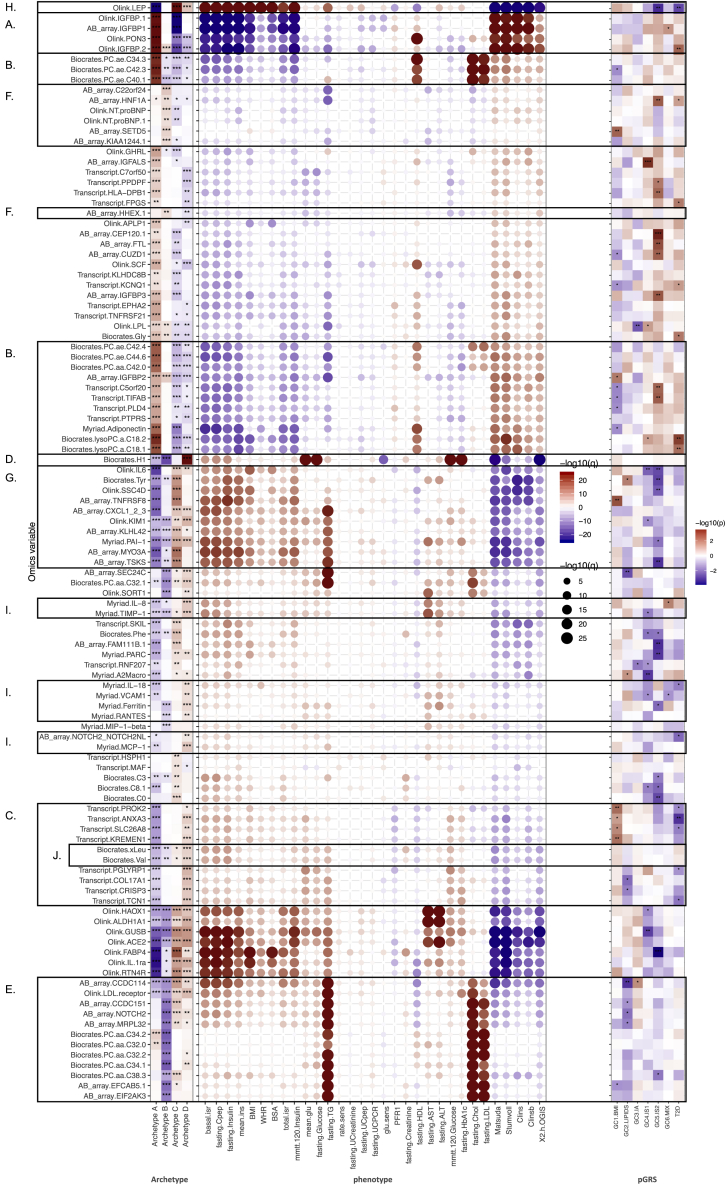
Summary of differences in multiomics profiles among the archetypes (A) Omics signatures discriminating archetype A (lean and insulin deficient) and archetype C (obese and insulin resistant) were associated with increased protein levels of insulin-like growth factor binding proteins 1 and 2 (IGFBP1 and IGFBP2). These proteins were positively associated with insulin-sensitivity-related variables. (B) Archetype A was further associated with increased metabolite levels of acyl-alkyl-phosphatidylcholines (PC.ae) that, in addition to insulin sensitivity, were positively associated with total cholesterol, HDL-C, and LDL-C levels; lyso-phosphatidylcholines (lysoPCs); and adiponectin (positively associated with HDL-C levels). (C) Omics signatures discriminating between archetype A (lean and insulin deficient) and archetype D (global severe) included transcript levels of several genes associated with insulin resistance and glycemic control. (D) Metabolite hexose (H1) was strongly negatively associated with archetype B (obese and insulin sensitive) and positively associated with archetype D, which were associated with the best and worst glucose control, respectively. (E) Biomarker levels negatively associated with archetype B were strongly positively associated with TG, total cholesterol, and LDL-C levels and include the proteins NOTCH2 and the LDL-C receptor, as well as metabolites and short-chained diacyl-phosphatidylcholines. (F) Protein levels positively associated with archetype B included biomarkers with weaker associations to the clinical phenotypes, such as the β cell marker HNF1A, which was negatively associated with TG levels. (G) Protein levels positively associated with archetype C included tyrosine and were positively associated with insulin resistance and TG levels and negatively associated with HDL-C. (H) Adipose tissue-derived hormone leptin (LEP) was strongly associated with the insulin-resistant obese phenotype represented by archetypes C and D. (I) Levels of inflammatory proteins discriminated between archetype D and archetype A/B and were positively associated with ALT and AST. (J) Branched-chain amino acids (BCAAs) valine and leucine/isoleucine discriminated between archetype D and archetype A and were associated with insulin-resistance-related variables. We tested the association between the quantitative archetype scores and each omics variable in linear regression models. The most discriminative omics variables were then investigated for associations with the clinical phenotypes. Statistically significant differences are marked as ∗q < 0.05, ∗∗q < 0.01, and ∗∗∗q < 0.001.

We then investigated whether the observed dysfunction in the etiological processes can be inferred from circulating molecular profiles. We looked for proteins, metabolites, or genes significantly (<5% false discovery rate [FDR]) associated with each of the quantitative archetype scores (Table S5A–S5F), and we then investigated the associations of the most discriminative omics variables with the clinical phenotypes. We also investigated the top omics variables for associations with the pGRSs to elucidate whether these could have a causal role or they were secondary to the phenotypic features that characterized the archetypes (Figure 5). We found the highest number of discriminative omics features for archetype A (lean and insulin deficient), containing 348 proteins, 177 metabolites, and 3,356 genes. This included proteins also associated with increased insulin sensitivity, such as insulin-like growth factor binding protein (IGFBP) 1 and IGFBP2 (q < 3.2 × 10^−25^)^14^^,^^15^ and paraoxonase 3 (PON3, q = 1.4 × 10^−30^) (Figure 5A). PON3 was positively associated with high-density lipoprotein cholesterol (HDL-C). Higher levels of IGFBP2 and IGFBP3 were nominally associated with increased T2D-GRS and IS2-pGRS, respectively, suggesting that these proteins were genetically linked to defective β cell function. We found several acyl-alkyl-phosphatidylcholines (PC.ae, q < 9.7 × 10^−16^), lyso-phosphatidylcholines (lysoPCs, q < 7.8 × 10^−14^), and adiponectin (q = 3.6 × 10^−15^)^16^ positively associated with the archetype A score and associated with high HDL-C, low-density lipoprotein cholesterol (LDL-C), and total cholesterol (Figure 5B). The lysoPCs were nominally associated with T2D-GRS. We also found several blood transcripts associated with both archetype A and archetype D, but with opposite directions, and not specifically associated with clinical phenotypes. Many of these transcripts were nominally associated with a low T2D-GRS and/or a high BMI-pGRS (Figure 5C). Through enrichment of immune cell signatures, we found that these represented B and T cell transcripts associated with archetype A and neutrophil transcripts associated with archetype D (Figure S3C). This finding likely represents the differences in the neutrophil-to-lymphocyte ratio, used as an inflammatory biomarker with prognostic value in several disease areas, including cardiovascular diseases and cancers.^17^ The strongest omics signatures for the score for archetype B (obese and insulin sensitive) were lower monosaccharides (H1, q = 4.3 × 10^−16^) (Figure 4D), in line with the best glucose control for this archetype, as well as lower shorter-chained diacyl-phosphatidylcholines (PC.aa, q < 7.7 × 10^−11^), lower protein levels of NOTCH2 (q = 3.1 × 10^−13^), and a lower LDL-C receptor (q = 1.1 × 10^−8^) linked to lower lipid levels (Figure 5E). There was no pGRS association for the monosaccharides, indicating that this signature was reactive to the glucose control. Four proteins linked to lipid levels, including NOTCH2, were inversely associated with the LIPID-pGRS, which suggests that low levels of these proteins were associated with high LIPID-pGRS and a higher risk of dyslipidemia. We found a group of β cell-linked proteins associated with a high archetype B score, including HNF1A and HHEX (q = 0.001) (Figure 5F), through an exploratory proteomics analysis. HNF1A protein levels were nominally associated with higher IS2-pGRS and T2D-GRS.

Omics signatures of archetype C (obese and insulin resistant) were largely those seen for archetype A (lean and insulin deficient), with the opposite direction of effect. They recapitulated the insulin-resistant phenotype of this archetype and included previously reported markers of this phenotype: tyrosine (Tyr, q = 1.1 × 10^−10^)^18^ and a group of proteins associated with TG levels, including CXCL1 (q = 7.5 × 10^−6^), PAI-1 (4.3 × 10^−8^), and MYO3A (q = 3.0 × 10^−14^) (Figure 5G). Many biomarkers of this group were nominally associated with lower IS2-pGRS, in line with the negative association observed between archetype C and IS2-pGRS. We also observed an association with higher protein levels of the adipose-tissue-derived hormone leptin (q = 2.9 × 10^−41^) (Figure 5H), in line with the obesity association in this archetype.^19^ Leptin levels were nominally associated with lower IS2-pGRS and lower T2D-GRS, again in line with the genetic association for archetype C. Leptin was also associated to archetype D, although to a lesser extent compared with archetype C.

Finally, omics signatures of archetype D (global severe) included inflammatory markers, such as interleukin (IL)-8, IL-18, TIMP-1, and MCP-1 (q < 1.1 × 10^−3^), as well as ferritin (q < 3.7 × 10^−3^), associated here with the liver enzymes ALT and AST (Figure 5I). We also noted markers of higher T2D risk,^20^ i.e., the branched-chain amino acids (BCAAs) valine (Val) and isoleucine (xLeu, q < 3.8 × 10^−3^), among the markers of this archetype (Figure 5J); however, none of them had strong associations with the pGRSs.

### Mixed etiology of archetypes shows precedence over clinical phenotypes

**Figure 6:**
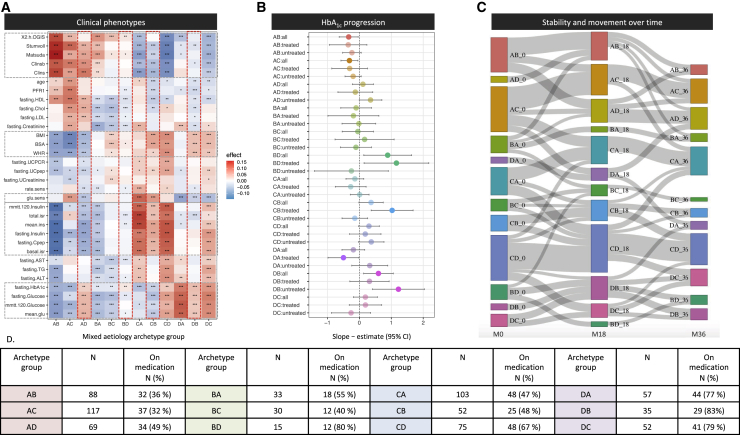
Characterization of the 12 mixed etiology archetypes (A) Heatmap of clinical associations across the 12 mixed etiology groups defined by their primary and secondary archetypes. The color of the heatmap reflects the directionality and magnitude of the test estimate, with red indicating higher and blue indicating lower values characteristic of the given group. Statistical significance is marked as ∗p < 0.05, ∗∗p < 0.01, and ∗∗∗p < 0.001. Gray stippled horizontal boxes highlight processes in which an archetype has precedence over other archetypes. Red vertical stippled boxes highlight mixed etiology archetypes associated with higher progression rates (see B). (B) Association between mixed etiology archetypes and disease progression as defined by the slope of HbA_1c_ over 36 months. The analysis was divided into all individuals, untreated individuals, and individuals treated with glucose-lowering medication at baseline for each group. Error bars represent 95% confidence intervals. (C) Sankey chart of movements among mixed etiology archetypes from baseline visit (M0), 18 months (M18), and 36 months (M36). Only trajectories followed by ≥5 participants are displayed for readability. (D) Table showing the number of individuals in the mixed etiology archetype groups and their frequency of glucose-lowering treatment at baseline.

Although the clinical presentation of individuals at the extremes of the phenotype distribution is captured by the characteristics of their high-scoring archetype, for most individuals represented collectively as the large mixed etiology group, we observe contributions of secondary (and tertiary) archetypes. To illustrate this, we examined how the combination of primary and secondary archetypes for individuals affected their clinical presentation. The heatmap in Figure 6A shows that there was agreement with the phenotype characteristics of the primary archetype within each of 12 mixed archetype groups: AB, AC, AD, BA, BC, BD, CA, CB, CD, DA, DB, and DC. However, it also shows that some archetypes had precedence over others in specific combinations. Archetype A as a primary or secondary archetype was associated with low BMI and waist-to-hip ratio (WHR), whereas archetype B as a primary or secondary archetype was associated with low lipids. Archetype C was associated with hyperinsulinemia and insulin resistance, but only when it was the primary archetype or combined with archetype D. Individuals with archetype D as the primary and secondary archetypes had high glucose and HbA_1c_ levels and low glucose sensitivity. Archetypes C and D were associated with high versus low glucose sensitivity, but archetype D appeared to be driving this, because the DC archetype group was associated with low glucose sensitivity. Figure 6B shows how the mixed archetypes combined to associate with HbA_1c_ progression. The results aligned well with the quantitative archetype analysis and show that groups with archetype A as the primary or secondary archetype trend toward a slower progression rate, except when archetype A was combined with archetype D as a secondary archetype. Groups with archetype D as the primary or secondary archetype showed trends toward faster progression, except when combined with archetype A. The highest progression rates were seen for groups DB, BD, and CB. If we distinguished between treated and untreated individuals within each group, we saw that two groups stood out as having higher progression when on glucose-lowering treatment: BD and CB, which for BD most likely was explained by 80% of individuals being on treatment. CB was associated with faster progression despite having low HbA_1c_ and glucose levels and high glucose sensitivity, and 48% of were being on glucose-lowering treatment, which indicates that the treatment might not be effective for this group. In addition, any group containing archetype D as the primary or secondary archetype also had the highest rates of glucose-lowering medications, in line with the main results.

### Archetype stability at follow-up

Stability over time was investigated for the 12 mixed etiology archetypes using the archetype group assignments at 0, 18, and 36 months. For this purpose, we analyzed 1,740 individual visits comprising 726, 591, and 423 individuals at the three visits, respectively, and divided the participants into mixed archetype groups at each visit, as described earlier. The Sankey chart in Figure 6C shows the flow among groups over time. We included flows with ≥5 individuals in the plot to ease the readability. This analysis shows that the mixed etiology archetypes consisting of archetypes A, C, and D were stable over time, with most individuals moving within the groups composed of the same primary archetype. Archetype groups composed of archetype B were the least stable; however, most individuals in an archetype-B-dominating group move either to the same group or to a group with the primary archetype inverted (BA → AB, BC → CB, etc.). The DA group also showed this tendency between month 18 and month 36.

## Discussion

The phenotypic landscape of individuals newly diagnosed with T2D forms a continuum of degrees of dysfunction within biological processes contributing to the complex etiology. Despite the common notion that the disease presentation at diagnosis is highly heterogeneous, it has been challenging to identify specific T2D endotypes with clearly defined boundaries. Therefore, instead of partitioning the dataset into discrete hard clusters as attempted before by others,^5^^,^^7^^,^^21^ we applied a soft-clustering method to detect the archetypes that capture the continuous combinations of dysfunction in the underlying etiological processes for individuals. This approach identified the combinatorial effect of five etiological processes: insulin secretion (archetype A), obesity (archetypes B, C, and D), insulin resistance (archetypes C and D), dyslipidemia (archetypes C and D), and reduced β cell glucose sensitivity (archetype D).

As already indicated by others,^5^ the differences in genetic profiles among archetypes provide evidence that the disease presentation is driven partly by differences in genetics. In our study, the pGRS analysis helped disentangle some primary defects associated with each archetype score. Functional studies of loci associated with T2D risk have revealed that a large portion of the loci exert their function through β cell dysfunction, which might bias our pGRS analysis toward this signature.^22^^,^^23^^,^^24^ Archetype A, which was associated with insulin-sensitivity-related traits, was positively associated with the two reduced insulin secretion (IS) pGRSs. It seems likely that the relatively high insulin sensitivity that is seen in individuals from this group represents an artifact arising from the ascertainment of those with newly diagnosed, relatively well-controlled diabetes. One consequence of this may have been that the individuals with the most marked insulin deficiency were, as a group, characterized by relatively well-preserved insulin action (reflected in better-than-average insulin sensitivity). Thus, individuals with a high score for archetype A appear more insulin sensitive and less obese compared with others. The composite phenotypes captured by archetype A are mediated by a different genetic risk, such as lower BMI-pGRS, or environmental factors, including increased exercise and lower calorie intake. Circulating omics signatures for archetype A recapitulated the insulin-sensitive phenotype, such as the IGFBP proteins.

On the other end of the spectrum are individuals with a high score for archetype C (obese and insulin resistant) who have developed diabetes despite lower overall genetic risk, largely reflecting lower genetic load for β cell dysfunction (low IS-pGRSs). We speculate that their T2D must be primarily driven by insulin resistance. The insulin resistance in this case was not captured by genetic risk for obesity or insulin resistance; therefore, it was likely largely driven by environmental exposures. The top circulating biomarkers for archetype C were leptin and tyrosine, both associated with obesity and insulin resistance.

Archetype D (global severe) was not associated with any distinct underlying genetic mechanism. Nevertheless, individuals with a high score for archetype D had the worst metabolic dysfunction, manifested by high liver fat, dyslipidemia, and high diastolic blood pressure. We further found that a high archetype D score was associated with the fastest disease progression. This was most significant among the participants not receiving glucose-lowering medication, suggesting that early identification of individuals characterized by archetype D and adjusting their medication regime could be beneficial. Indeed, we observed the highest likelihood of participants on T2D medication for this archetype; hence, evaluating additional molecular biomarkers of this archetype may be valuable to facilitate early detection. Our study suggests that this archetype had particularly high levels of inflammatory markers, triglycerides, and branched-chain amino acids.

Archetype B (obese and insulin sensitive) was associated with higher T2D risk because of greater obesity, corroborated by high BMI-pGRS. The obese, insulin-sensitive phenotype was associated with a favorable lipid profile, indicating a more metabolically healthy obesity profile. Intriguingly, we found β cell function markers in circulation (HNF1A and HHEX proteins) to be associated with this archetype and, in this case, associated with lower blood lipid levels, prompting the need for validation. In support of the favorable lipid profiles, we found archetype B to be negatively associated with markers linked to TG, total cholesterol, and LDL-C (such as shorter-chained PC.aa and the LDL receptor).

We performed a detailed characterization of the groups displaying mixed etiology by varying degrees of contributions from primary and secondary archetypes and provided examples of how defining softer cut points allowed us to explore the mixed category but also highlighted the challenge of choosing more cut points to dissect out this group that was truly of mixed etiology. The clinical associations for the mixed archetype groups helped us dissect out phenotypes for which some archetypes have precedence over others in specific combinations. We saw that archetype A controlled lower BMI and WHR over archetype C, which in turn controlled high insulin levels and high glucose sensitivity. Archetype D dominated high glucose and HbA_1c_ levels and low glucose sensitivity, whereas archetype B controlled low lipid and creatinine levels. Translating the phenotypic patterns directly to effects on disease progression proved to be challenging. There does not seem to be a single pattern of phenotypes that control the progression rate; rather, there was complex interplay between the mixed etiology archetypes and the level of treatment within each group.

### Limitations of study

Limitations of our study include the difficulty of disentangling which of the phenotypes were the main drivers of the archetypes and which were secondary due to ascertainment effects. We used genetics to try to assign the causality. Likewise, we reported numerous omics signatures associated with the archetypes and again used genetics to tease out whether these could be causal. However, these associations depended on the phenotypes linked to the archetypes and should be confirmed in independent cohorts before their usefulness as prognostic markers can be fully evaluated. External validation in an independent cohort of the findings reported here has not been possible, because no similar studies have this detailed level of phenotyping available. In addition, it would be valuable to have a longer follow-up period to assess the effect of the archetypes on the development of diabetes-related complications. We acknowledge that the size of the group with mixed etiology was large compared with the individuals at the extreme. The soft-clustering approach showed that there is phenotypic variation among individuals, which has some structure, but that it is best resolved in the archetype extremes. We explore the molecular, physiological, and phenotypic variation to provide insight into the heterogeneity of type 2 diabetes, but we do not think one should try to over-interpret the data to infer clinical utility.

Nevertheless, the detailed and harmonized longitudinal phenotyping of the IMI DIRECT cohort, in combination with a method that capture the continuous combination of etiological processes, helps redefine the disease mechanisms for which the identified omics signatures serve as proxies. Better understanding of these signatures’ contribution to the T2D phenotypes will be imperative to apply them to stratify patients and guide future treatment decisions.

## STAR★Methods

### Key resources table

**Table:** 

REAGENT or RESOURCE	SOURCE	IDENTIFIER
**Critical commercial assays**		

Myriad proteomics panel	Myriad	https://rbm.q2labsolutions.com/
OLINK proteomics panel	OLINK	https://www.olink.com/
Biocrates targeted metabolomics panel	Biocrates	https://biocrates.com/
Metabolon untargeted metabolomics panel	Metabolon	https://www.metabolon.com/

**Deposited data**		

Antibody bead array proteomics	This study	DIRECTdataaccess@Dundee.ac.uk
Myriad proteomics panel	This study	DIRECTdataaccess@Dundee.ac.uk
OLINK proteomics panel	This study	DIRECTdataaccess@Dundee.ac.uk
Biocrates targeted metabolomics panel	This study	DIRECTdataaccess@Dundee.ac.uk
Metabolon untargeted metabolomics panel	This study	DIRECTdataaccess@Dundee.ac.uk
Whole blood RNAseq transcriptomics	This study	DIRECTdataaccess@Dundee.ac.uk
Clinical and anthropometry	This study	DIRECTdataaccess@Dundee.ac.uk
Biochemical	This study	DIRECTdataaccess@Dundee.ac.uk
MRI	This study	DIRECTdataaccess@Dundee.ac.uk
Glycemic modeling	This study	DIRECTdataaccess@Dundee.ac.uk
Accelerometry	This study	DIRECTdataaccess@Dundee.ac.uk
Diet questionnaire	This study	DIRECTdataaccess@Dundee.ac.uk

**Software and algorithms**		

R version 3.4.0	https://cran.r-project.org/	N/A
Archetype R package	Eugster and Leisch, 2009. DOI:10.18637/jss.v030.i08.;Eugster and Leisch, 2011	N/A

### Resource availability

#### Lead contact

Further information should be directed to the Lead Contact, Søren Brunak (soren.brunak@cpr.ku.dk).

#### Materials availability

This study did not generate any new reagents.

### Experimental models and subject details

We focus exclusively on the newly-diagnosed sub-cohort of the IMI-DIRECT study, consisting of 789 participants identified through general practice and other registers, as described previously.^25^ The mean age at inclusion was 62 years with the youngest 35 years at baseline, which should exclude any individuals with MODY. Participants were diagnosed within two years before recruitment, were on lifestyle and/or metformin treatment only, and had glycated haemoglobin (HbA_1c_) < 60.0 mmol/mol (< 7.6%) within previous three months. A total of 726 participants with complete baseline data were retained. Complete follow-up data was available for 591 participants at 18 months, and 423 at 36 months of the study. Detailed descriptions of the IMI-DIRECT study cohorts have been reported previously.^25^^,^^26^ Approval for the study protocol was obtained from each of the regional research ethics review boards separately (Lund, Sweden: 20130312105459927, Copenhagen, Denmark: H-1-2012-166 and H-1-2012-100, Amsterdam, Netherlands: NL40099.029.12, Newcastle, Dundee and Exeter, UK: 12/NE/0132) and all participants provided written informed consent at enrolment. The research conformed to the ethical principles for medical research involving human participants outlined in the declaration of Helsinki.

### Method details

#### Baseline phenotypes

Thirty-two clinical phenotypes were selected for inclusion in the clustering analysis, all of which were measured at baseline, 18 months and 36 months follow-up (Table 1). The properties of measurements and assays for these variables have been described previously.^25^^,^^26^ Age, gender, height, weight, body-mass-index (BMI) and waist-hip-ratio (WHR) were collected at each visit. Variables measured in fasting plasma included glucose, insulin, C-peptide, total cholesterol, HDL-C and LDL-C cholesterol, triglycerides (TG), creatinine, ALT and AST. A frequently-sampled mixed-meal tolerance test (MMTT) following a 250 mL liquid drink (Fortisip: 18.4 g carbohydrate per 100 ml) was performed at each visit from which measures of glucose and insulin dynamics were calculated for 2h oral glucose insulin sensitivity (2h OGIS), Stumvoll and Matsuda sensitivity indices, mean glucose and insulin, basal and total insulin secretion, β-cell glucose sensitivity, potentiation factor ratio, rate sensitivity, and insulin clearance (described in next section). Measurements in urine included urine C-peptide/creatinine ratio (UCPCR), C-peptide and creatinine. HbA_1c_ was measured at baseline and at 9, 18, 27, and 36 months.

#### Glycaemic modeling

Beta-cell function was assessed from the MMTT using a model that describes the relationship between insulin secretion and glucose concentration, which has been illustrated in detail previously.^27^^,^^28^ The model expresses insulin secretion rate (in pmol·min^-1.^m^-2^) as the sum of two components. The first component represents the dependence of insulin secretion on absolute glucose concentration at any time point during the MMTT through a dose-response function relating the two variables. Characteristic parameters of the dose-response are the mean slope over the observed glucose range, denoted as β-cell *glucose sensitivity*, and *insulin secretion at a fixed glucose concentration* of 8 mmol/L (approximately average fasting glucose at baseline). The dose-response is modulated by a *potentiation factor*, which accounts for the fact that during an acute stimulation insulin secretion may be higher on the descending phase of hyperglycaemia than on the ascending phase at the same glucose concentration. As such, the potentiation factor encompasses several potentiating mechanisms (prolonged exposure to hyperglycaemia, non-glucose substrates, gastro-intestinal hormones, neural modulation, drug effects). It is set to be a positive function of time, and is constrained to average unity during the experiment. In normal subjects, the potentiation factor typically increases from baseline to the end of a 2-hour MMTT.^29^ To quantify this excursion, the ratio between the 2-hour and the baseline value was calculated. This ratio is denoted as *potentiation factor ratio*. The second insulin secretion component represents the dependence of insulin secretion on the rate of change of glucose concentration. This component is related to the glucose derivative component (for derivative component > 0), and is determined by a single parameter, denoted as *rate sensitivity*. Rate sensitivity is related to early insulin release.^29^

The model parameters were estimated from glucose and C-peptide concentrations by regularized least-squares, as previously described.^27^ Regularization involves the choice of smoothing factors, which were selected to obtain glucose and C-peptide model residuals with standard deviations close to the expected measurement error (∼1% for glucose and ∼4% for C-peptide). Insulin secretion rates were calculated from the model every 5 min. The integral of insulin secretion during the 2-hour MMTT represents the total insulin secretion.

#### Additional phenotypes used to characterize archetypes

The following variables were not complete for all 726 subjects, or were only measured at baseline, and were therefore not included in the clustering analysis but only used to characterize the archetypes (see Table S3 for number of observations). Plasma concentrations of total and active GLP-1 were measured at 0 and 60 min during the MMTT, glucagon was measured at 0, 60 and 120 min, and stimulated pro-insulin at 60 min. The volume of abdominal adipose tissue was measured in liters using magnetic resonance imaging (MRI), reported as trunk fat, visceral fat and subcutaneous abdominal fat. Liver and pancreas fat and iron were derived simultaneously.^30^^,^^31^ Quantitative measures of physical activity were derived from triaxial accelerometers.^25^ Glutamic acid decarboxylase antibody (GADA) measurements were available for all participants at baseline. GADA positive subjects comprised only ∼1.5% (n = 11) of the individuals included, and was in line with the use of the 97.5^th^ centile laboratory threshold for GADA positivity. These 11 patients did not stand out in terms of other measured phenotypes, therefore we decided to include them in the analysis with the remaining participants.

#### Disease progression

Diabetes progression was assessed by change in HbA_1c_ over time using individual HbA_1c_ slopes. HbA_1c_ concentrations were measured at baseline and 9, 18, 27 and 36 months after start of the study. HbA_1c_ trajectories were described with a conditional linear mixed-effect model.^32^ The conditional approach employs a linear transformation of the data and produces a longitudinal and a cross-sectional component, which are orthogonal. The transformation makes modeling of the longitudinal component, which is relevant for HbA_1c_ trajectories, independent of the cross-sectional effects, which are potential confounders and were not considered. In particular, the approach eliminates possible spurious correlations between the longitudinal parameters and baseline HbA_1c_, which may arise if baseline HbA_1c_ is not accurately modeled. Transformed HbA_1c_ was modeled according to a mixed-effect approach as the sum of the following terms:

• a proportional effect of time, estimated via the parameter *slope*_*i*_, where *i* represents a specific individual, represented as a random variable with a normal distribution;
• a proportional effect of BMI;
• a linear effect of the metformin dose, expressed as percentage of a maximal dose of 3 g;
• a linear effect of the cumulative dose for the other anti-diabetic drugs (insulin excluded), expressed as sum of the percentages of the maximum dose of each drug;
• a constant effect of insulin treatment;
• a proportional effect of delay in HbA_1c_ assay, i.e., of the difference between the assay and the sampling times;
• a residual error *ε*_*ik*_, where *k* refers to the time point, represented as a random variable from a normal distribution with zero mean.

The insulin and BMI effects were constrained to be negative and positive, respectively. The linear effects of the treatment *dose* were 0 for *dose* = 0 and *a*+*b*∙*dose* for *dose* > 0, where *a* and *b* were different for metformin and the other drugs and were constrained to be negative. A medication was considered effective at a given time if it was taken at least 30 days before. The *slope*_*i*_ parameter represents the HbA_1c_ underlying progression, adjusted for changes in BMI and antidiabetic treatments. Subjects were included in the analysis if at least two HbA_1c_ values were available (n = 696). The model was estimated using Monolix 2016 R1 (Lixoft. MONOLIX, https://lixoft.com/products/monolix/).

#### Archetype soft-clustering

All variables were rank-normally transformed and residualized for XX/XY genotype and recruitment center at each time-point using linear regression analysis. We performed soft-clustering using the R package ‘archetypes’,^11^^,^^12^ with ‘robustArchetypes’ function, and selected the combination of baseline archetypes which best minimized the residual sum of squares over 100 iterations of the algorithm. We evaluated the number of archetypes that best fitted this dataset by multiple approaches. First, we assessed the minimized residual sum of squares (RSS) for *k* number of archetypes ranging from 1 to 10 in a scree plot (Figure 1A). Then, we assessed the stability of the *k* archetypes by a randomized subsampling of 90% of the original dataset repeated 100 times (Figure 1B) using the adjusted Rand index. Simultaneously, we evaluated the stability of the archetypes at archetype membership thresholds from 0.0 to 1.0 in intervals of 0.05 (Figures 1B and 1C). Together these analyses showed that four archetypes had the lowest RSS while showing high stability after randomization. The subgroup stability increased with increasing membership threshold and plateaued at 0.6: we therefore used this threshold to define extreme archetype-scores. All individuals with memberships < 0.6 for all of the archetypes were assigned to the ‘mixed etiology’ group (i.e., no membership to any extreme archetype, n = 472). We performed a post hoc Silhouette analysis with a standard distance matrix for the four extreme achetype groups and the mixed etiology group using the *factoextra* package in R. This analysis showed that the four extreme archetype groups look well clustered, and that the individuals in the mixed etiology group do not form a homogeneous cluster (Figure S1).

#### Mixed etiology groupings

To explore further the group with mixed etiology we divided the participants based on the highest and second highest scoring archetype. Out of the 472 participants in the mixed etiology group 306 had a single dominating archetype (score > = 0.4). Two dominating archetypes (score > = 0.4) were found in 68 individuals and 98 did not have any dominating archetype (all archetype scores < 0.4). The mixed etiology archetypes were divided into 12 groups based on their primary and secondary archetypes, and were analyzed for association with the clinical phenotypes, HbA_1c_ progression and stability at follow-up time points.

#### Parameter pruning

To see if there was a smalled set of clustering parameters that could recover most of the information in the full set of phenotypes we pruned the clustering input phenotypes by pairwise Pearson correlation. We constructed pruned datasets by removing parameters correlated at a progressively lower correlation coefficient, starting at 0.8 and ending at 0.2. This removed 10 to 28 of the original 32 parameters. We then ran archetype clustering for each pruned dataset and evaluated the cosine similarity between the original archetypes and the archetypes at each pruning. We next identified archetype similarity > 0.8 at each pruning and searched for a subset where this cut-off was fulfilled for all four original archetypes. This resulted in a dataset that was pruned at a correlation coefficient cut-off at 0.6 and that retained 15 of the original parameters (Figure S2A). A heatmap of the resulting associations between the 32 input parameters and the pruned archetypes at correlation 0.6 is shown in Figure S2B and compared to the original heatmap in Figure S2C.

#### DNA extraction, genotyping, and quality control

DNA extraction of participants at high risk of diabetes and new onset of diabetes was carried out using Maxwell 16 Blood DNA purification kits and a Maxwell 16 semi-automated nucleic acid purification system (Promega). Genotyping was conducted in two tranches using the Illumina HumanCore array (HCE24 v1.0) and genotypes were called using Illumina’s GenCall algorithm.

Samples were excluded for any of the following reasons: call rate < 97%; low or excess mean heterozygosity; gender discordance; duplicates; and monozygosity. Genotyping quality control was then performed to provide high-quality genotype data for downstream analyses using the following criteria: call rate < 99%; deviation from Hardy-Weinberg equilibrium (exact p < 0.001); variants not mapped to human genome build GRCh37; and variants with duplicate chromosome positions.

We performed an additional quality control step to identify plausible sample swaps and/or sample labeling errors utilizing available RNaseq data on genotyped samples. For each sample with both genotype and RNaseq data availables, we identified the best matching expression-genotype pair using expression profile and genotypes (see transcriptomics section below). When mismatches between genotype and expression samples were identified, we examined the reported and (genotype) derived gender and traced back the samples through each step involved in their acquisition, extraction, and genotyping and re-mapped the samples to correct identifiers.

We carried out a second round of genotyping of 96 samples to: (i) confirm the correct assignment of the matched DNA/RNA samples; and (ii) recover genotyped samples that failed quality control due to low genotype rate. We repeated quality assessment of these samples as described above and then combined samples from both genotype tranches and conducted another round of sample and variant quality control using the same criteria as above. We also confirmed the correct alignment for all DNA/RNA samples fixed above. We used autosomal variants with MAF > 1% that passed quality control to construct axes of genetic variation using principal components analysis implemented in PLINK software to identify ethnic outliers defined as non-European ancestry using the 1000 Genomes Project samples as reference.^33^ A total of 795 European samples with genotype-RNaseq pairing passed the final quality control.

#### Pre-phasing and imputation

All samples passing quality control were taken forward for pre-phasing and imputation. Before pre-phasing variants were removed if: (i) allele frequencies differed from those for European ancestry haplotypes from the 1000 Genomes reference panel by more than 20%; (ii) AT/GC variants had MAF > 40% because of potential undetected errors in strand alignment; or (iii) MAF < 1% because of difficulties in calling rare variants. After these exclusions, a total of 273,568 variants remained. Samples were first pre-phased using SHAPEIT1 (version v2.r790) and then imputed up to the 1000 Genomes Project reference panel (phase 3, October 2014 release; X chromosome, phase 3, August 2015 release) using IMPUTEv2.3.^34^^,^^35^

#### Construction of genetic risk scores

Genetic risk scores (GRS) were constructed as the weighted sum of the risk alleles over all leading variants at 403 independent signals associated with risk of T2D^1^ in the European population for the overall T2D GRS. Partitioned GRSs associated with reduced insulin secretion coupled with high proinsulin (IS1) and low proinsulin (IS2), insulin action (IA), adiposity (BMI), dyslipidemia (LIPID), and mixed features (MIX) were constructed in a similar manner using leading variants at loci associated with the T2D-related quantitative traits in other GWAS cohorts, as described previously.^1^^,^^9^^,^^13^ A table describing the pGRSs and which loci they were constructed from is available in Figure S4.

#### Sequences generation

For the study of transcriptomic profiles samples of mRNA from whole blood samples were processed for RNA-sequencing. Concentration of mRNA per samples was assessed using the Qubit2.0 from Invitrogen. The quality of the samples was then assessed using the TapeStation Software (A.01.04) with an RNA Screen Tape from Agilent to check the mRNA quality on gel and samples were discarded due to low mRNA quality. The remaining samples were processed and sequencing libraries were prepared. Quality of the libraries was evaluated using Qubit and TapeStation using DNA1000 Screen Tape. One sample was discarded after library preparation due to low quality. The remaining samples were placed in Flow cell PE using the cBOT system from Illumina. The samples were then sequenced on the Illumina HiSec2000 platform using 49 bp paired-end reads.

#### Read mapping and exon quantifications

The 49-bp sequenced paired-end reads were mapped to the GRCh37 reference genome^36^ with GEMTools 1.7.1.^37^ Exon quantifications were calculated for all elements annotated on GENCODE v19.^38^ All overlapping exons of a gene were merged into meta-exons with identifier of type ENSG000001.1_exon.start.pos_exon.end.pos. Read counts over these elements used paired-end reads if their both ends have a quality score > = 150, a total mismatch ≤ 5 (5 mismatches max in 2x49pb) and if they are in proper orientation. We filtered transcripts from genes that were not protein coding, lincRNA or processed transcripts if they overlap in the opposite strand with protein coding genes and lincRNA genes. For split reads, we counted the exon overlap of each split fragment, and added counts per read as 1/(number of overlapping bases per exon). For genes quantification, FPKM values were calculated.

#### Samples quality assessment and filtering

Samples with a total number of exonic reads lower than 5e+06 reads or with a proportion of exonic reads over the total number of reads lower than 20% were considered of low quality excluded.

Identification of samples mix-ups and labeling errors is possible when genotypes are available.^39^ For each sample with genotyping data available, we tested the heterozygous sites in DNA genotypes for expression of both alleles in the RNaseq data. Samples mix-ups or mislabeled show lower levels of expression of both alleles. Using the function *match* from the suite QTLtools,^40^ we tested each expression profile (BAM files) against all imputed genotypes from DIRECT to identify the best matching expression-genotype pair (as described above for genotyping data).

Samples were removed for low quality or being mixes of RNaseq samples that could not be identified with confidence and if no suitable match between expression and the available genotypes were found. After correcting samples swaps, 3 individuals were found to have duplicated RNaseq data. To confirm the correct assignment of the matched DNA/RNA samples and recovered failed genotypes during QC we re-genotyped samples from 96 individuals. After repeating the genotypes calling and quality assessment, we confirmed the correct alignment for those samples. We also recovered samples for which genotypes were not available in the first round.

Gender identification in RNaseq compare expression levels of genes in the autosomal region for the chromosome Y and the expression of the *XIST* gene in the chromosome X. To confirm gender information and validate the identity of the sequence data, we compare the gender provided by clinical reports and the gender identified by genotype data with the gender identified from RNaseq data and samples with inconclusive gender analysis in expression were due to small mixes of RNaseq samples, low RNaseq quality or un-reported biological factors.

#### Filtering

Genes and exons with more than 50% of zero reads were removed from the study. To ensure enough individuals with no zero reads in the study we filter those exons and genes with zero reads in more than 50% individuals as defined by the cohort at screening. Finally, exons and genes from chromosome Y, mitochondria, and level 3 annotation, as encoded by Gencode v19, were removed from further analysis.

#### Software

At the time of the study, custom scripts were used for any intermediate step and quantification of exon and genes, as well as quality assessment of the samples. The same pipeline can now be found in Delaneau et al.^41^ as part of QTLtools.

#### Enrichment of transcriptomics signatures in immune cells

Enrichment of nominally associated (p < 0.05) transcriptomics signatures of extreme archetype scores described in this study for transcriptomic signatures of immune cell types was performed using the Human Immune Cell Transcriptome dataset (GSE3982) obtained from the NCBI Gene Expression Omnibus (GEO). Using the online NCBI GEO2R tool, we performed differential expression analysis comparing each cell type to all other immune cell types within the dataset (Basophils, Mast Cells, Eosinophils, Dendritic cells, Macrophages, Neutrophils, B cells, Effector memory T cells, NK cells, Central memory T cells, Th1 cells, Th2 cells). The log2-fold change-ranked gene lists formed the comparative signatures of the immune cell types. We tested the enrichment of module genes within these ranked gene lists using the ‘gage’ generally applicable gene-set enrichment Bioconductor package.^42^

#### Targeted metabolomics

Plasma concentrations of 163 metabolites were determined using a FIA-ESI-MS/MS-based targeted metabolomics approach with the Absolute *IDQ*™ p150 kit (BIOCRATES Life Sciences AG, Innsbruck, Austria). The assay allows simultaneous quantification of 163 metabolites out of 10 μL plasma, and includes free carnitine, 40 acylcarnitines (Cx:y), 15 amino acids (Leu and Ile are measured togetheras xLeu), hexoses (sum of hexoses – about 90%–95% glucose), 91 glycerophospholipids (15 lysophosphatidylcholines (lysoPC.Cx:y) and 76 phosphatidylcholines (PC.Cx:y)), and 15 sphingolipids (SM.Cx:yc). The abbreviations Cx:y are used to describe the total number of carbons and double bonds of all chains, respectively.

The LODs were set to three times the values of the zero samples (PBS). The LLOQ and ULOQ were determined experimentally by Biocrates. The assay procedures of the Absolute*IDQ*™ p150 kit as well as the metabolite nomenclature have been described in detail previously.^43^^,^^44^ Analytical specifications for LOD and evaluated quantification ranges, further LOD for semiquantitative measurements, identities of quantitative and semiquantitative metabolites, specificity, potential interferences, linearity, precision and accuracy, reproducibility, and stability were described in Biocrates manual AS-P150.

Sample handling was performed by a Hamilton Microlab STAR™ robot (Hamilton Bonaduz AG, Bonaduz, Switzerland) and a Ultravap nitrogen evaporator (Porvair Sciences, Leatherhead, UK), beside standard laboratory equipment. Mass spectrometric analyses were done on an API 4000 triple quadrupole system (Sciex Deutschland GmbH, Darmstadt, Germany) equipped with a 1200 Series HPLC (Agilent Technologies Deutschland GmbH, Böblingen, Germany) and a HTC PAL auto sampler (CTC Analytics, Zwingen, Switzerland) controlled by the software Analyst 1.6.2. Data evaluation for quantification of metabolite concentrations and quality assessment was performed with the software MultiQuant 3.0.1 (Sciex) and the Met*IDQ* software package, which is an integral part of the Absolute*IDQ* kit. Metabolite concentrations were calculated using internal standards and reported in μM. In addition to the investigated samples, five aliquots of a pooled reference plasma (Ref_Plasma-Hum_PK3) were analyzed on each kit plate. These reference plasma samples were used for normalization purposes and for calculation of coefficient of variance (CV) for each metabolite.

#### Quality control

After data export from Met*IDQ*^*TM*,^ a first technical QC comprising analysis of peak shapes, retention times, and compound identity was performed. In a second QC step, possible batch effects and effects of different phenotypes were investigated using principal component analysis (PCA). Data were corrected for batches. Lower outliers were defined as samples with > 33% of metabolite concentrations below 25% quantile – 1.5∗IQR. Upper outliers were defined as samples with > 33% of metabolite concentrations above 25% quantile + 1.5∗IQR. Metabolite traits with too many zero concentration samples and NAs (> 50%) were excluded (none). The Coefficient of Variation (CV) was calculated in reference samples for each metabolite over all plates. Metabolite traits with CV > 0.25 were excluded. Metabolite traits with > 95% of samples below LOD were marked.

#### Untargeted Metabolomics

Plasma samples were stored at −80°C prior to analysis at Helmholtz Zentrum München, Germany. On the day of extraction, samples were thawed on ice, were randomized, and were distributed into 25 batches for the T2D cohort. A hundred μL of the plasma were pipetted into a 2 mL 96-well plate. In addition to samples from this study, a pooled human reference plasma sample (Seralab, West Sussex, UK) was extracted in the same way as samples of the study and placed on 7 wells of each batch. These samples served as technical replicates throughout the dataset to assess process variability. Besides those samples, 100 μL of water was extracted as samples of the study and placed in 6 wells of each 96-well plate to serve as process blanks.

Protein was precipitated and the metabolites in the plasma samples were extracted with 475 μL methanol, containing four recovery standard compounds to monitor the extraction efficiency. After centrifugation, the supernatant was split into 4 aliquots of 100 μL each onto two 96-well microplates. The first 2 aliquots were used for LC-MS/MS analysis in positive and negative electrospray ionization mode. Two further aliquots on the second plate were kept as a reserve. The samples were dried on a TurboVap 96 (Zymark, Sotax, Lörrach, Germany). Prior to LC-MS/MS in positive ion mode, the samples were reconstituted with 50 μL of 0.1% formic acid and those analyzed in negative ion mode with 50 μL of 6.5 mM ammonium bicarbonate, pH 8.0. Reconstitution solvents for both ionization modes contained further internal standards that allowed monitoring of instrument performance and also served as retention reference markers. To minimize human error, liquid handling was performed on a Hamilton Microlab STAR robot (Hamilton Bonaduz AG, Bonaduz, Switzerland).

LC-MS/MS analysis was performed on a linear ion trap LTQ XL mass spectrometer (Thermo Fisher Scientific GmbH, Dreieich, Germany) coupled with a Waters Acquity UPLC system (Waters GmbH, Eschborn, Germany). Two separate columns (2.1 × 100 mm Waters BEH C18 1.7 μm particle) were used for acidic (solvent A: 0.1% formic acid in water, solvent B: 0.1% formic acid in methanol) and for basic (A: 6.5 mM ammonium bicarbonate pH 8.0, B: 6.5 mM ammonium bicarbonate in 95% methanol) mobile phase conditions, optimized for positive and negative electrospray ionization, respectively. After injection of the sample extracts, the columns were developed in a gradient of 99.5% A to 98% B in 11 min run time at 350 μL/min flow rate. The eluent flow was directly connected to the ESI source of the LTQ XL mass spectrometer. Full scan mass spectra (80 – 1000 m/z) and data dependent MS/MS scans with dynamic exclusion were recorded in turns. Metabolites were annotated by curation of the LC-MS/MS data against proprietary Metabolon’s chemical database library (Metabolon, Inc., Durham, NC, USA) based on retention index, precursor mass and MS/MS spectra. In this study, 544 metabolites, 341 compounds of known identity (named biochemical) and 203 compounds of unknown structural identity (unnamed biochemical) were identified. The unknown chemicals are indicated by a letter X followed by a number as the compound identifier.

#### Antibody and target selection

A Biomarker Task Force was formed with the DIRECT consortium to select proteins of interested for plasma analysis. This led to a list of 442 protein candidates found with associations with diabetes found in previous studies using literature mining, protein and gene expression in beta cells and islets, proteins of clinical relevance, GWAS and eQTL studies, previous use of antibodies in the applied assay, as well as a concluding network analysis. Antibodies then chosen based on availability from the Human Protein Atlas (HPA).^45^^,^^46^^,^^47^ We found 779 HPA antibodies for 385 out of 442 proteins. Prioritizing the proteins, for which more than one antibody is accessible, 640 antibodies for 252 proteins were chosen for antibody performance tests. The antibodies were applied to assays with a subset of 340 plasma samples using the assay procedure as described below to test the property of the antibodies in the contexts of these samples. Antibodies were excluded from further studies if 1) signal intensities were obtained lower than the internal negative control (6 HPAs were excludes) and 2) the variance in signal intensities across samples were smaller than the control antibody (127 HPAs were excluded). A set of 380 antibodies were selected for subsequent analyses that target the 265 proteins.

#### Generation of antibody bead arrays

All selected HPA antibodies were coupled to beads to generate antibody bead arrays in suspension (as described below). As assay controls, antibodies against albumin (DAKO) and anti-human IgG (Jackson ImmunoResearch) were included, as well as beads coupled with normal rabbit IgG to resemble the scaffold of HPA antibodies. One bead identity did not include any protein during the coupling procedure (denoted bare beads).

Antibodies were coupled to carboxylated magnetic beads (MagPlex-C, Luminex Corp.) in accordance to previously developed protocols.^48^^,^^49^^,^^50^ Briefly, 5 × 10^5^ beads per bead identity were distributed in 96-well microtiter plates (Greiner BioOne). Beads were initially washed and re-suspended in phosphate buffer (0.1 M NaH2PO4, pH 6.2) using a plate washer (EL406, Biotek). Bead activation was performed by adding 0.5 mg 1-ethyl-3(3-dimethylamino-propyl) carbodiimide (Pierce) and 0.5 mg N-hydroxysuccinimide (Pierce) dissolved in 100 μl phosphate buffer. After 20 min incubation at 650 rpm on a plate shaker (Grant Bio), beads were washed with 0.1 M 2-(N-morpholino)ethanesulfonic acid (MES) buffer (pH 4.5) on a plate washer (EL406, Biotek). 1.6 μg of each antibody had been pre-diluted in 100 μl of MES buffer by a liquid handling system (EVO150, Tecan) and were subsequently added to the activated beads. After 2 h incubation at RT, beads were washed 3 × in PBS-T (1 × PBS, 0.05% Tween20). Next, 50 μl of a protein blocking buffer (Blocking Reagent for ELISA, Roche Applied Science) supplemented with 0.1% (v/v) ProClin (Sigma-Aldrich) was added for an overnight incubation at 4°C. Finally, mixing the 384 different bead identities resulted in 384-plexed suspension bead arrays that were stored at 4°C in the dark until further use. R-Phycoerythrin-conjugated donkey anti-rabbit IgG antibody (Jackson ImmunoResearch) was utilized to confirm an efficient coupling of antibodies.

#### Experimental design

Samples from the four different centers were distributed across microtiter plates via a supervised randomization procedure. The plate layouts were carefully designed to minimize and equalize the time that each sample was placed at room temperature during the transferring of samples into plates. To achieve this, plasma samples were in designated orders, thawed over night at 4°C, centrifuged for 10 min at 3,000 × g, and distributed into the designed plate layout by the use of a liquid handling system (Freedom EVO150, TECAN). After sample randomization, the randomized 96-well microtiter plates were stored at −80°C until further use.

#### Antibody beads array assays

Plasma samples in randomized plate layouts were thawed at 4°C and centrifuged for 10 min at 3,000 × g. Three microliters of each sample were diluted in 22 μl of 1x PBS using a liquid handler (SELMA, CyBio). Biotinylation of diluted plasma was performed as previously described.^49^ Briefly, labeling was enabled by a 2 h incubation of samples with a 10-fold molar excess of NHS-PEG4-Biotin (Pierce) at 4°C. The biotinylation reaction was quenched by the addition of 0.5 M Tris-HCl (pH 8.0) with a 250-fold molar excess over biotin. After 20 min incubation with Tris-HCl at 4°C, samples were stored at −80°C until usage.

#### Biotinylated samples were diluted 1:50 using a liquid handler (SELMA, CyBio) in assay buffer.

The assay buffer was composed of 0.5% (w/v) polyvinylalcohol and 0.8% (w/v) polyvinylpyrrolidone (Sigma) in 0.1% (w/v) casein (Sigma-Aldrich) in PBS (PVXC) supplemented with 0.5 mg/ml rabbit IgG (Bethyl). Prior incubation with beads, samples were heat-treated at 56°C for 30 min in a water bath (TW8, Julabo) followed by 15 min cooling at RT. 5 μl of the antibody suspension bead array (∼200 beads per bead identity) was distributed into 384-well microtiter plates (Greiner BioOne). 45 μl of heat-treated samples were then added to each bead plate by the use of a liquid handler (SELMA, CyBio). After an overnight incubation at RT on a shaking table (Grant) beads were washed with 3 × 50 μl PBS-T on a plate washer (EL406, Biotek). Samples were cross-linked with 0.4% paraformaldehyde in PBS-T for 10 min, washed 3 × 50 μl PBS-T and 50 μl of 0.5 lg/ml R-phycoerythrin labeled streptavidin (Invitrogen) in PBS-T was added. After 20 min incubation, beads were finally washed 3 × 50 μl PBS-T and resuspended in 50 μl PBS-T for measurement in a FlexMap3D instrument (Luminex Corp.). At least 50 bead counts were counted per bead identity. The median fluorescence intensity (MFI) was used to represent the relative amount of target protein binding to each of the antibody-coupled bead identity.

#### Data quality assessment

The obtained data was evaluated based on intensity levels and three antibodies were excluded from further analysis as the median MFI were below negative control antibodies (bare and rabbit IgG beads). Because one stock solution of mixed beads was created and aliquoted into each assay plate, other experimental errors were linked to the procedure for individual samples. Thus, eight samples were flagged that seemingly failed. Such samples were those 1) that had median values of MFIs ± 2 SD or below the median of control measurement without any sample (buffer only), and 2) that were identified as outliers using Robust PCA using ‘rrcov’ R package (version 1.4-3).^51^ The cutoff probability values in an outlier diagnostic plot were set to 0.001 for both score and orthogonal distances. The samples deviating beyond the cutoffs in both distance coordinates were classified as outliers, setting alpha, the proportional tolerance, to 0.9. The remaining dataset was denoted as annotated.

#### Data pre-processing

The annotated data was processed as by PQN^52^ for sample-by-sample variation within the samples collected in same center and assay plates analyzed on the same day. The variation introduced by multiple assay plates was minimized by Multi-MA normalization.^53^ Inverse normal transformation was then applied to the normalized data to reduce the effects of outliers.

#### Plasma Proteomics – targeted assays

Samples from DIRECT study centers were manually randomization by a mix-shake-distribute procedure and placed into 96-well plates. All samples were analyzed at SciLifeLab in Stockholm using several different immunoassay platforms.

Proteins were measured in EDTA plasma using the Cardiometabolic, Cardiovascular II, Cardiovascular III, Development and Metabolism panels from Olink Proteomics AB (Uppsala, Sweden) according to the instructions for the Proximity Extension Assays (PEA).^54^ The obtained normalized expression values (NPX) values were obtained from Olink’s NPX manager software version 0.0.85.0. Magnetic bead-based assays were used for the analysis of FGF21 (SPRCUS627, MerckMillipore) and a panel consisting of For CXCL10, ICAM-1, IL1R-alpha, and RETN (LXSAHM, R&D Systems). The assays were performed according to the instructions and the instrumentation for liquid handling as introduced above. The beads were analyzed using the FlexMap 3D instrument (Luminex Corp.) operated by the xPONENT software version 4.2. The obtained MFI values were converted into concentration values using 5-parametric fitting. Plasma levels of IL1-beta and TNFR1-alpha were quantified in accordance with the instructions for the microfluidic ELISA assays^55^ from ProteinSimple. Additional proteins were analyzed in randomized samples using the services from Myriad RBM (Myriad GmbH, Germany).

#### Archetype stability at follow-up

To assess how the participants’ phenotypic presentation differed at 18 and 36 months follow-up visits, we collected all phenotype data from the baseline visit and the two follow-up visits. We rank-normally transformed the data together, and residualized for XX/XY genotype and recruitment center as described previously. We performed the archetypes soft-clustering with four archetype-scores as decribed above, and evaluated the stability by calculating the pairwise pearson correlation between all archetype-scores across the three time-points. We hypothesized that the stability could depend on the location of an individual along the axis of the archetype-scores at baseline, and therefore, also calculated and compared the mean archetype-scores across time-points in each group.

### Quantification and statistical analysis

#### Clinical variables

We tested the association of archetype scores evaluated at month 0 with all continuous phenotypes using linear regression. In addition, we also tested the differences between the groups with extreme archetype scores (membership threshold 0.6) using the Kruskal-Wallis test, and compared each group individually to the remaining individuals using the Mann-Whitney U test.

#### Disease progression

Differences in glyceamic deterioration were evaluated by investigating the slope of change in HbA_1c_ over time. This analysis was stratified into participants who did and did not receive any glucose-lowering medication during the course of this study. We assessed the associations of all individual phenotypes, as well as the collective archetype scores, with the disease progression using linear regression. Additionally, we tested whether the individual likelihood of receiving glucose-lowering medication at each of the time points differed between archetype scores using logistic regression. Similarly, we tested the likelihood of individuals either starting a new treatment or increasing the current dosage of the glucose-lowering medication during the course of the study. Statistical significance was set at p < 0.05.

#### Statistical analysis of circulating omics variables and genetic risk scores

All circulating omics variables were residualized for age, XX/XY genotype and recruitment center.

We tested each of the omics variables (16,209 transcripts, 732 proteins, 357 metabolites, and 7 GRSs) separately using linear regression with each archetype score. The effect of metformin treatment at baseline on the omics results was investigated by two sensitivity approaches 1) running the linear regression on the subset of individuals who did not receive metformin separately, and 2) correcting for the use of metformin in the regression model. We compared the results for the top associated features to the original uncorrected results and saw that this did not alter the results for the omics associations significantly. The p values were adjusted for multiple testing using the Benjamini-Hochberg procedure for decreasing the false discovery rate (FDR) for each omics dataset separately. A q < 0.05 was considered statistically significant. Additionaly, the genetic association between the top omics biomarkers was tested for each of the six partitioned GRSs and the T2D GRS separately using linear regression. All statistical analyses were performed in R/3.4.0.

## Consortia

The members of the IMI DIRECT Consortium are Leen 't Hart, Moustafa Abdalla, Jonathan Adam, Jerzey Adamski, Kofi Adragni, Rosa LundbyeAllesøe, Kristine Allin, Manimozhiyan Arumugam, Naeimeh Atabaki Pasdar, Tania Baltauss, Karina Banasik, Patrick Baum, Jimmy Bell, Margit Bergstrom, Joline Beulens, Susanna Bianzano, Roberto Bizzotto, Amélie Bonnefond, Caroline Anna Brorsson, Andrew Brown, Søren Brunak, Louise Cabrelli, Robert Caiazzo, Mickaël Canouil, Matilda Dale, David Davtian, Adem Dawed, Federico De Masi, Nathalie de Preville, Koen Dekkers, Emmanouil Dermitzakis, Harshal Deshmukh, Christiane Dings, Louise Donnelly, Avirup Dutta, Beate Ehrhardt, Petra Elders, Line Engelbrechtsen, Rebeca Eriksen, Yong Fan, Juan Fernandez, Jorge Ferrer, Hugo Fitipaldi, Ian Forgie, Annemette Forman, Paul Franks, Francesca Frau, Andreas Fritsche, Philippe Froguel, Gary Frost, Johann Gassenhuber, Giuseppe Giordano, Toni Giorgino, Stephen Gough, Ulrike Graefe-Mody, Harald Grallert, Rolf Grempler, Lenka Groeneveld, Leif Groop, Valborg Gudmundsdóttir, Ramneek Gupta, Mark Haid, Torben Hansen, Tue Hansen, Andrew Hattersley, Ragna Haussler, Alison Heggie, Anita Hennige, Anita Hill, Reinhard Holl, Mun-Gwan Hong, Michelle Hudson, Bernd Jablonka, Christopher Jennison, Yunlong Jiao, Joachim Johansen, Angus Jones, Anna Jonsson, Tugce Karaderi, Jane Kaye, Maria Klintenberg, Robert Koivula, Tarja Kokkola, Azra Kurbasic, Teemu Kuulasmaa, Markku Laakso, Thorsten Lehr, Heather Loftus, Agnete Troen Lundgaard, Anubha Mahajan, Andrea Mari, Gianluca Mazzoni, Mark McCarthy, Timothy McDonald, Donna McEvoy, Nicky McRobert, Ian McVittie, Miranda Mourby, Petra Musholt, Pascal Mutie, Rachel Nice, Claudia Nicolay, Agnes Martine Nielsen, Giel Nijpels, Birgitte Nilsson, Colin Palmer, Francois Pattou, Imre Pavo, Ewan Pearson, Oluf Pedersen, Helle Pedersen, Mandy Perry, Hugo Pomares-Millan, Anna Ramisch, Simon Rasmussen, Violeta Raverdi, Martin Ridderstråle, Neil Robertson, Marianne Rodriquez, Hartmut Ruetten, Femke Rutters, Nina Scherer, Jochen Schwenk, Nisha Shah, Sapna Sharma, Iryna Sihinevich, Roderick Slieker, Nadja Sondertoft, Hans-Henrik Staerfeldt, Birgit Steckel-Hamann, Harriet Teare, Cecilia Engel Thomas, Melissa Thomas, Elizabeth Louise Thomas, Henrik Thomsen, Barbara Thorand, Claire Thorne, Joachim Tillner, Martina Troll, Konstantinos Tsirigos, Andrea Tura, Mathias Uhlen, Amber van der Heijden, Nienke van Leeuwen, Sabine van Oort, Jagadish Vangipurapu, Helene Verkindt, Henrik Vestergaard, Ana Viñuela, Josef Vogt, Peter Wad Sackett, Mark Walker, Agata Wesolowska-Andersen, Brandon Whitcher, Margaret White, and Han Wu.

## Data Availability

• Data: Requests for access to IMI DIRECT data, including data presented here, can be made to the Lead Contact. All data are available without restriction in a secure environment. • Code: Our manuscript does not report any novel custom code. The software for the main clustering method is available as an R package and was published in reference 10 and 11. • Any additional information required to reanalyze the data reported in this paper is available from the Lead Contact upon request.

## Supplemental information

Document S1. Figures S1–S5

Table S1. Association between clustering phenotypes and archetype scores A. Associations with groups of individuals with extreme archetype scores. B. Association with quantitative archetype scores. Related to Figure 2B and Figure S3A.

Table S2. Associations between partitioned T2D genetic risk scores and archetype scores, related to Figure 3A

Table S3. Associations between additional phenotypes (not included in the archetype clustering analysis) and archetype scores, related to Figure 3B

Table S4. Association between disease progression and archetype scores A. Assessed by the slope of HbA_1C_ during 36 months of follow-up. Related to Figure 4A. B. Change in treatment indicates either start of new treatment or increase in dose of existing treatment. Related to Figures 4C and 4D.

Table S5. Associations between omics biomarkers and archetype scores, related to Figure 5 A. antibody bead array proteomics. B. proteomics Myriad panel. C. proteomics OLINK panels. D. targeted metabolomics Biocrates panel. E. untargeted metabolomics Metabolon panel. F. whole blood RNA-seq trancriptomics.

Table S6. Associations between top omics biomarkers and pGRS, related to Figure 5

Document S2. Article plus supplemental information
