# Supplementary material for: Four groups of type 2 diabetes contribute to the etiological and clinical heterogeneity in newly diagnosed individuals: An IMI DIRECT study
Source: Cell Rep Med. 2022 Jan 4;3(1):100477. doi: 10.1016/j.xcrm.2021.100477 (PMC8784706; doi:10.1016/j.xcrm.2021.100477)
Supplement: Document S1. Figures S1–S5 [file mmc1.pdf]

Cell Reports Medicine, Volume *3*

Supplemental information

Four groups of type 2 diabetes contribute to the etiological and clinical heterogeneity in newly diagnosed individuals: An IMI DIRECT study

Agata Wesolowska-Andersen, Caroline A. Brorsson, Roberto Bizzotto, Andrea Mari, Andrea Tura, Robert Koivula, Anubha Mahajan, Ana Vinuela, Juan Fernandez Tajes, Sapna Sharma, Mark Haid, Cornelia Prehn, Anna Artati, Mun-Gwan Hong, Petra B. Musholt, Azra Kurbasic, Federico De Masi, Kostas Tsirigos, Helle Krogh Pedersen, Valborg Gudmundsdottir, Cecilia Engel Thomas, Karina Banasik, Chrisopher Jennison, Angus Jones, Gwen Kennedy, Jimmy Bell, Louise Thomas, Gary Frost, Henrik Thomsen, Kristine Allin, Tue Haldor Hansen, Henrik Vestergaard, Torben Hansen, Femke Rutters, Petra Elders, Leen t'Hart, Amelie Bonnefond, Mickaël Canouil, Soren Brage, Tarja Kokkola, Alison Heggie, Donna McEvoy, Andrew Hattersley, Timothy McDonald, Harriet Teare, Martin Ridderstrale, Mark Walker, Ian Forgie, Giuseppe N. Giordano, Philippe Froguel, Imre Pavo, Hartmut Ruetten, Oluf Pedersen, Emmanouil Dermitzakis, Paul W. Franks, Jochen M. Schwenk, Jerzy Adamski, Ewan Pearson, Mark I. McCarthy, Søren Brunak, and IMI DIRECT Consortium

Supplemental figures and tables

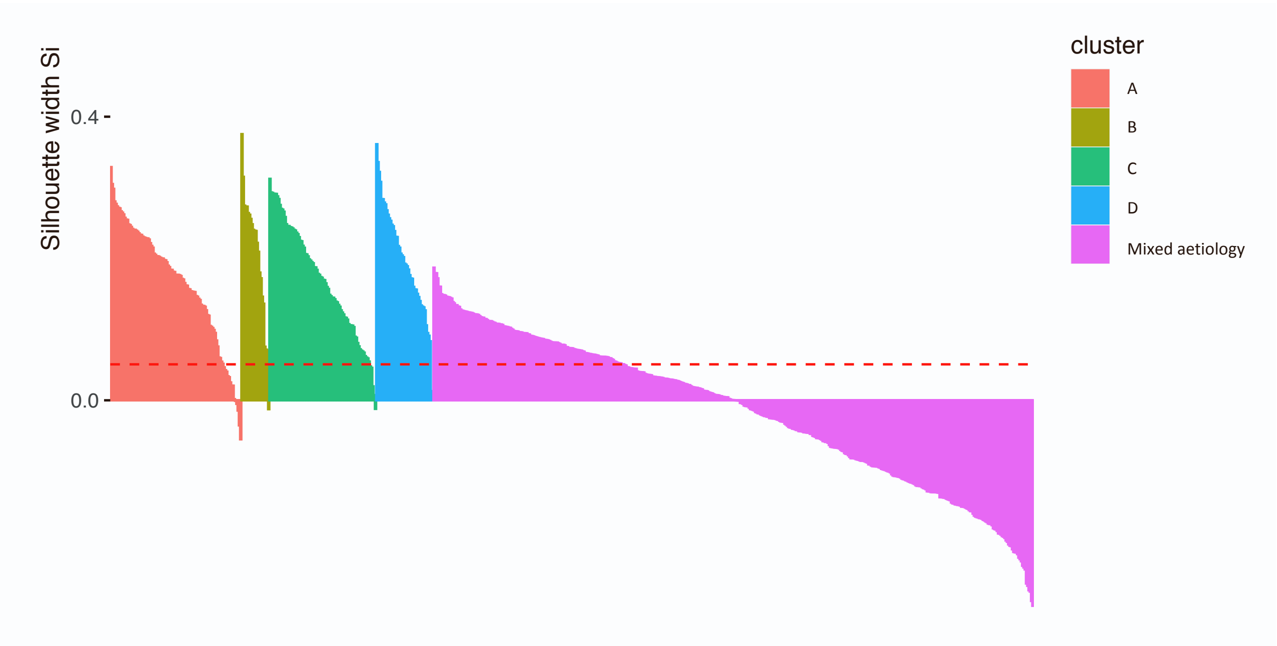

Figure S1. Silhouette analysis of clustering for extreme archetype individuals and mixed aetiology group. The silhoutette analysis showed that individuals in with extreme archetype values (membership > 0.6) for each of the four archetypes are well clustered. The mixed aethiology group do not form a homogeneous cluster. **Related to Figure 1 and Figure 6.**

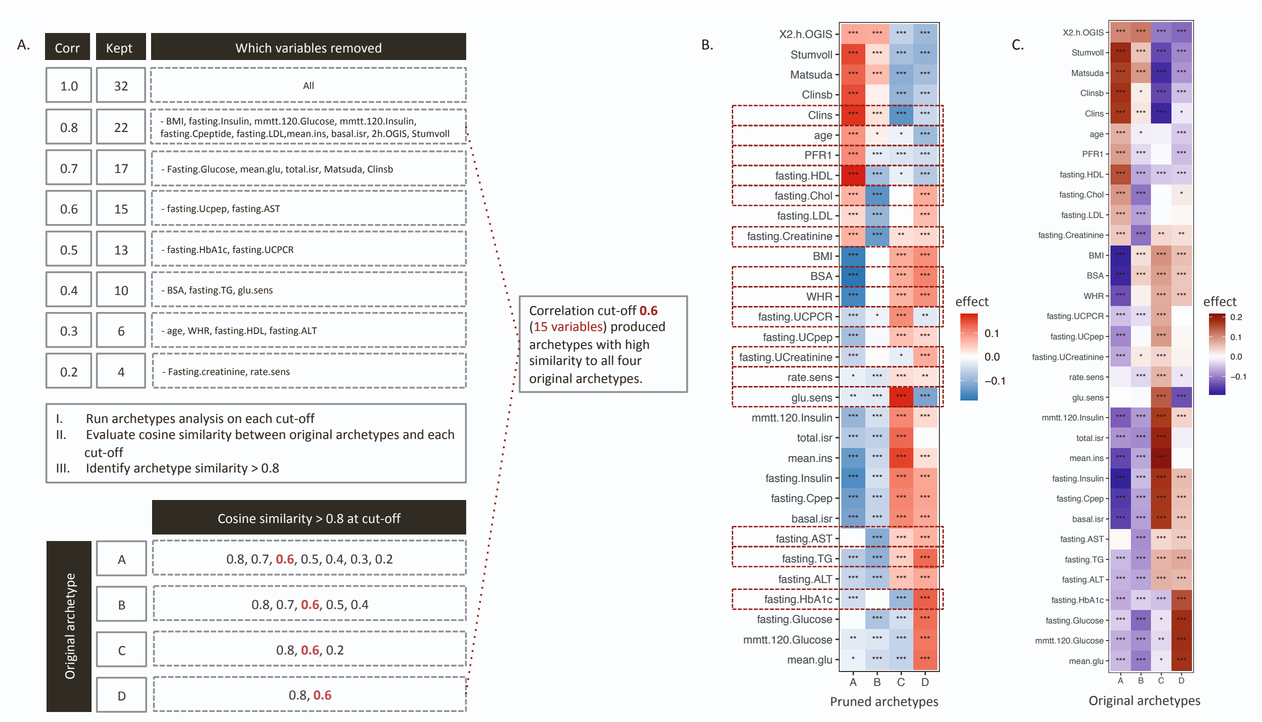

Figure S2. Pruning of clustering input data to find subset of variables which capture majority of the signal in the original archetype scores.
A. The input data set var pruned by recursively removing variables that met a cut-off based on Pearson correlation coefficients between 0.8 – 0.2 in steps of 0.1. Archetype clustering analysis was then run on each of the data set cuts according to the protocol for the original archetypes. For each cut we evaluated the cosine similarity with the original archetype scores and searched for a cut-off where all four archetypes were reconstructed at a cosine similarity > 0.8.

B. Heatmap showing the associations between all 32 clustering variables produced by the archetypes at correlation cut-off 0.6. This cut-off produced archetypes with high similarity to the original archetypes for all four archetype scores and resulted in a data set that retained 15 of the original 32 input variables for clustering (highlighted in red).

C. Heatmap of the associations between the 32 clustering variables and the original archetype scores for comparison with the pruned data.
* p<0.05, ** p<0.01, *** p<0.001

Related to STAR Method section "Parameter pruning".

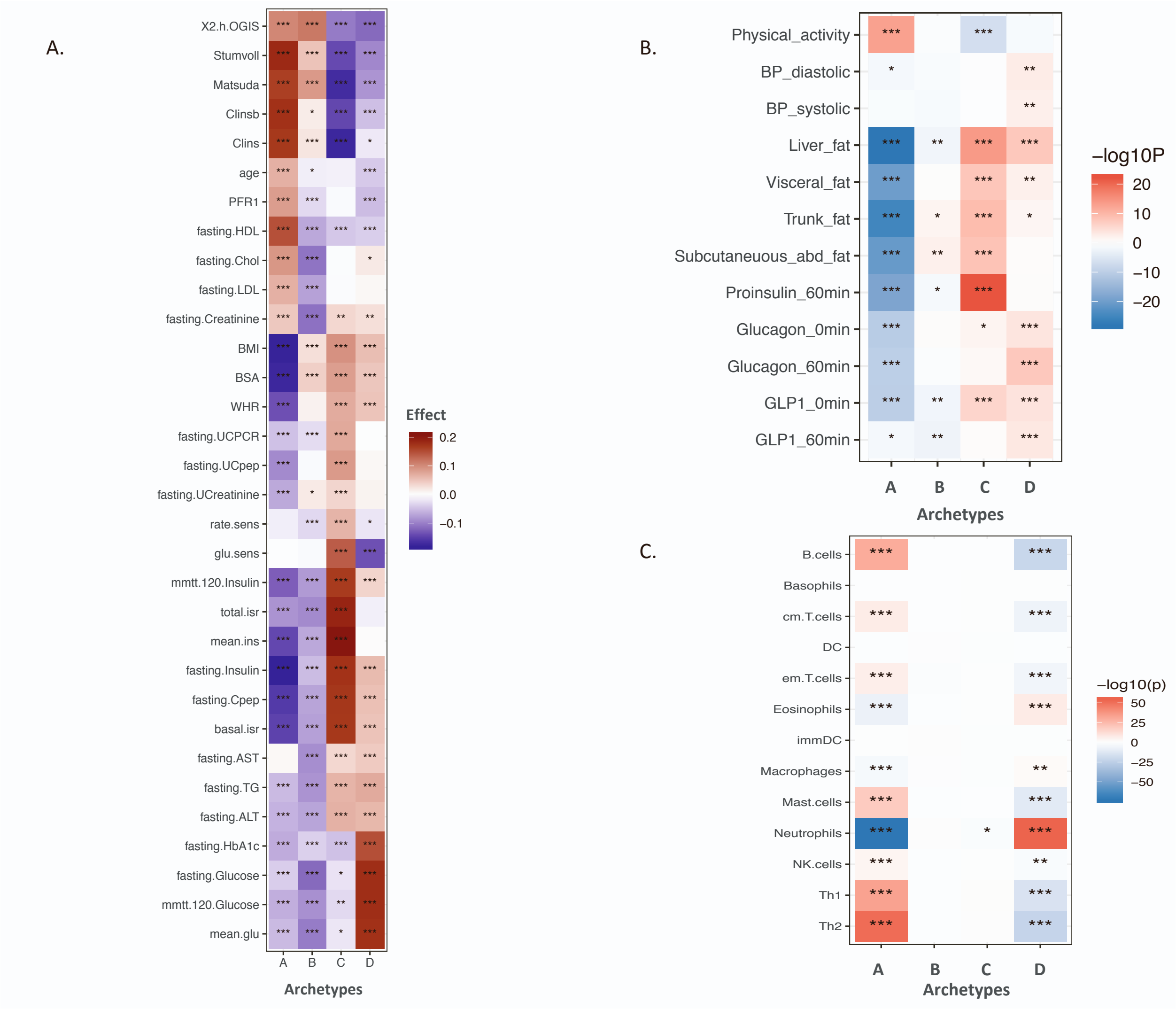

Figure S3. Heatmaps showing the associations between archetypes and the clustering variables and additional variables at baseline.

A. Heatmap of clustering variables shows highly similar results to the analysis using the extreme archetypes based on the 0.6 membership threshold. Associations were analysed by linear regression models for each of the archetype scores in the full cohort.

B. Heatmap showing the associations between the individuals with extreme archetype scores and additional variables at baseline. These include clinical, biochemical, physical activity, and MRI-based variables. Differences between subgroups were assessed by Mann-Whitney U test for each extreme group against the individuals in the remaining groups.

C. Enrichment of transcriptomics signatures in immune cells for groups of individuals with extreme archetype scores. Differences between groups were assessed by Mann-Whitney U test for each group against the individuals in the remaining groups.
* $p<0.05$, ** $p<0.01$, *** $p<0.001$
Related to figure 2.

A.

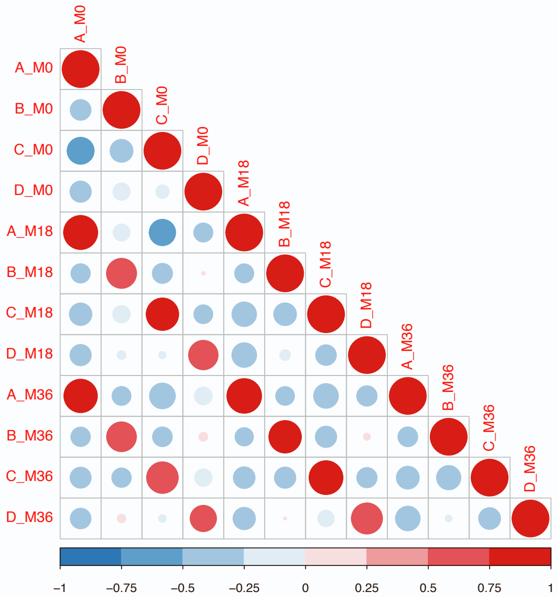

B.

	M0				M18				M36			
**Group**	**A**	**B**	**C**	**D**	**A**	**B**	**C**	**D**	**A**	**B**	**C**	**D**
**A**	**0.73**	0.09	0.10	0.08	**0.64**	0.08	0.14	0.14	**0.64**	0.09	0.15	0.12
**B**	0.11	**0.71**	0.11	0.07	0.14	**0.40**	0.24	0.23	0.11	**0.43**	0.19	0.27
**C**	0.07	0.08	**0.73**	0.11	0.07	0.06	**0.67**	0.20	0.09	0.06	**0.64**	0.21
**D**	0.02	0.19	0.10	**0.69**	0.03	0.15	0.20	**0.62**	0.06	0.10	0.10	**0.74**
**mix**	0.27	0.23	0.29	0.21	0.27	0.20	0.27	0.27	0.25	0.21	0.27	0.26

Figure S4. Archetypes stability at follow-up.
A. Correlation plot showing the Pearson correlation coefficient within and between archetype scores at baseline (M0), 18 months (M18) and 36 months (M36).

B. Tables showing mean of all archetypes across time-points divided by belonging to the groups with extreme archetype scores or the mixed aetiology group at baseline. **Related to figure 6C.**

pGRS name	Physiological impact		Loci included
**Insulin secretion 1 (IS1)**	Adverse impact on beta-cell function	High proinsulin	*ABO, ADCY5, GCK, HNF1A, MTNR1B, SLC30A8, TCF7L2, TMEM258*
**Insulin secretion 2 (IS2)**		Low proinsulin	*ADAMTS9, ANK1, C2CD4A-B, CCND2, CDKAL1, CDKN2A-B, CENTD2, DGKB, GLIS3, GPSM1, HHEX-IDE, HMG20A, IGF2BP2, JAZF1, KCNJ11, KCNQ1, KLHDC5, PROX1, THADA, ZBED3, ZHX3*
**Insulin action (IA)**	Reduced insulin sensitivity	Mediation via fat distribution	*ANKRD55, ARL15, BPTF, CMIP, FAM13A, GRB14, HNF4A, IRS1, KIF9, KLF14, LPL, MACF1, PEPD, PLCB3, PPARG, VEGFA*
**Adiposity (BMI)**		Mediation via obesity	*FTO, MC4R, NFAT5, NRXN3, POC5, TFAP2B*
**Dyslipidemia (LIPIDS)**		Mediation via lipid metabolism	*GCKR, TM6SF2, TOMM40_APOE*
**Mixed (MIX)**	Undetermined		*BCAR1, BCL11A, CDC123_CAMK1D, CENPW, CEP68, FAM63A, GIPR, HMGA2, HNF1A, HNF1B, HNF4A, HORMAD2, MHC, MLX, MPHOSPH9, MRAS, MTMR3, PAM, PAX4, PIM3, PLEKHA1, PNPLA3, PRC1, PTPN9, RREB1, SPRY2, TLE1, TMEM154, TPCN2, TSPAN8, TTLL6, WFS1, WSCD2, ZMIZ1, ZZEF1*

Figure S5. Table of loci included in the construction of the partitioned genetic risk scores. Related to Figure S3A.

SUPPLEMENTAL TABLES

Table S1. Association between clustering phenotypes and archetype scores.

A. Associations with groups of individuals with extreme archetype scores. B. Association with quantitative archetype scores. **Related to Figure 2B and Figure S3A.**

Table S2. Associations between partitioned T2D genetic risk scores and archetype scores. Related to Figure 3A.

Table S3. Associations between additional phenotypes (not included in the archetypes clustering analysis) and archetype scores. Related to Figure 3B.

Table S4. Association between disease progression and archetype scores.

A. Assessed by the slope of HbA1C during 36 months of follow-up. **Related to Figure 4A.** B. Change in treatment indicates either start of new treatment or increase in dose of existing treatment. **Related to Figure 4 C-D.**

Table S5. Associations between omics biomarkers and archetype scores. Related to Figure 5.

A. antibody bead array proteomics. B. proteomics Myriad panel. C. proteomics OLINK panels. D. targeted metabolomics Biocrates panel. E. untargeted metabolomics Metabolon panel. F. whole blood RNA-seq trancriptomics.

Table S6. Associations between top omics biomarkers and pGRS. Related to Figure 5.
